# The class B heat shock factor HSFB1 regulates heat tolerance in grapevine

**DOI:** 10.1093/hr/uhad001

**Published:** 2023-01-04

**Authors:** Haiyang Chen, Xinna Liu, Shenchang Li, Ling Yuan, Huayuan Mu, Yi Wang, Yang Li, Wei Duan, Peige Fan, Zhenchang Liang, Lijun Wang

**Affiliations:** Beijing Key Laboratory of Grape Science and Enology and Key Laboratory of Plant Resources, Institute of Botany, The Chinese Academy of Sciences, Beijing 100093, China; China National Botanical Garden, Beijing 100093, China; University of the Chinese Academy of Sciences, Beijing 100049, China; Beijing Key Laboratory of Grape Science and Enology and Key Laboratory of Plant Resources, Institute of Botany, Chinese Academy of Sciences, Beijing 100093, China; China National Botanical Garden, Beijing 100093, China; University of Chinese Academy of Sciences, Beijing 100049, China; Beijing Key Laboratory of Grape Science and Enology and Key Laboratory of Plant Resources, Institute of Botany, Chinese Academy of Sciences, Beijing 100093, China; China National Botanical Garden, Beijing 100093, China; University of the Chinese Academy of Sciences, Beijing 100049, China; Department of Plant and Soil Sciences, University of Kentucky, Lexington, Kentucky 40546, USA; Key Laboratory of South China Agricultural Plant Molecular Analysis and Genetic Improvement and Guangdong Provincial Key Laboratory of Applied Botany, South China Botanical Garden, Chinese Academy of Sciences, Guangzhou 510650, China; Beijing Key Laboratory of Grape Science and Enology and Key Laboratory of Plant Resources, Institute of Botany, Chinese Academy of Sciences, Beijing 100093, China; China National Botanical Garden, Beijing 100093, China; University of the Chinese Academy of Sciences, Beijing 100049, China; Beijing Key Laboratory of Grape Science and Enology and Key Laboratory of Plant Resources, Institute of Botany, Chinese Academy of Sciences, Beijing 100093, China; China National Botanical Garden, Beijing 100093, China; Beijing Key Laboratory of Grape Science and Enology and Key Laboratory of Plant Resources, Institute of Botany, Chinese Academy of Sciences, Beijing 100093, China; China National Botanical Garden, Beijing 100093, China; Beijing Key Laboratory of Grape Science and Enology and Key Laboratory of Plant Resources, Institute of Botany, Chinese Academy of Sciences, Beijing 100093, China; China National Botanical Garden, Beijing 100093, China; Beijing Key Laboratory of Grape Science and Enology and Key Laboratory of Plant Resources, Institute of Botany, Chinese Academy of Sciences, Beijing 100093, China; China National Botanical Garden, Beijing 100093, China; Beijing Key Laboratory of Grape Science and Enology and Key Laboratory of Plant Resources, Institute of Botany, Chinese Academy of Sciences, Beijing 100093, China; China National Botanical Garden, Beijing 100093, China; Beijing Key Laboratory of Grape Science and Enology and Key Laboratory of Plant Resources, Institute of Botany, Chinese Academy of Sciences, Beijing 100093, China; China National Botanical Garden, Beijing 100093, China

## Abstract

Grape is a widely cultivated crop with high economic value. Most cultivars derived from mild or cooler climates may not withstand increasing heat stress. Therefore, dissecting the mechanisms of heat tolerance in grapes is of particular significance. Here, we performed comparative transcriptome analysis of *Vitis davidii* ‘Tangwei’ (heat tolerant) and *Vitis vinifera* ‘Jingxiu’ (heat sensitive) grapevines after exposure to 25°C, 40°C, or 45°C for 2 h. More differentially expressed genes (DEGs) were detected in ‘Tangwei’ than in ‘Jingxiu’ in response to heat stress, and the number of DEGs increased with increasing treatment temperatures. We identified a class B Heat Shock Factor, HSFB1, which was significantly upregulated in ‘Tangwei’, but not in ‘Jingxiu’, at high temperature. VdHSFB1 from ‘Tangwei’ and VvHSFB1 from ‘Jingxiu’ differ in only one amino acid, and both showed similar transcriptional repression activities. Overexpression and RNA interference of *HSFB1* in grape indicated that HSFB1 positively regulates the heat tolerance. Moreover, the heat tolerance of *HSFB1*-overexpressing plants was positively correlated to *HSFB1* expression level. The activity of the *VdHSFB1* promoter is higher than that of *VvHSFB1* under both normal and high temperatures. Promoter analysis showed that more TATA-box and AT~TATA-box *cis*-elements are present in the *VdHSFB1* promoter than the *VvHSFB1* promoter. The promoter sequence variations between *VdHSFB1* and *VvHSFB1* likely determine the *HSFB1* expression levels that influence heat tolerance of the two grape germplasms with contrasting thermotolerance. Collectively, we validated the role of *HSFB1* in heat tolerance, and the knowledge gained will advance our ability to breed heat-tolerant grape cultivars.

## Introduction

Heat stress is broadly considered a major determining factor affecting crop growth and development, limiting production and quality [1]. Photosynthesis is a sensitive physiological process affected by high temperature. High temperature rapidly inhibits photosynthesis by changing chloroplast structure, inactivating ribulose-1,5-bisphosphate carboxylase-oxygenase (Rubisco), decreasing the abundance of photosynthetic pigments, and damaging photosystem II [2, 3]. In addition, high temperature influences the stability of proteins and membranes, induces the accumulation of reactive oxygen species (ROS), and changes the production and signal transduction of plant hormones, resulting in transcriptomic reprogramming and metabolomic changes [4, 5]. Nonetheless, plants have also evolved complex and inter-connected signaling pathways and response mechanisms to high temperature [6].

Grapevines are valuable crops widely cultivated throughout the world [7–9]. However, during the growing season, grapevines often suffer from heat stress, that affects development and fruit metabolism, thus limiting grape yield and quality [10]. In the past, our research on grape responses to high temperature mainly focused on physiological and morphological changes [11–14]. With advances in high-throughput RNA sequencing technology, we now possess a powerful tool for investigating the global response of plants to heat stress. Liu *et al*. characterized some stress-related genes that encode transcription factors (TFs), antioxidant enzymes, heat shock proteins (HSPs), and glycolytic enzymes by analysing the transcriptomics and proteomics of *Vitis vinifera* ‘Cabernet Sauvignon’ under high temperature [15, 16]. Jiang *et al*. analysed the transcriptomics and proteomics of *V. vinifera* ‘Jingxiangyu’ grapevines exposed to 35°C, 40°C, and 45°C, and revealed that alternative splicing is an important post-transcriptional regulatory event during grapevine responses to heat stress [17]. Based on phosphoproteomic and acetylproteomic analyses of the ‘Jingxiangyu’ leaves under elevated temperature, phosphorylation of serine/arginine-rich splicing factors is involved in heat response [18]. By investigating the effects of high temperature on grape berries based on proteomic and metabolomics analyses, Lecourieux *et al*. found 592 differentially accumulated proteins in Cabernet Sauvignon plants [19]. However, previous research has mostly focused on heat-sensitive cultivars of *V. vinifera*, resulting in a limited understanding of grapevine responses to high temperatures. By analysing the transcriptional profiles of heat-tolerant and sensitive tea cultivars, 78 differential expressed genes (DEGs) have been identified [20], validating a rational approach to identify the key genes using two varieties with contrasting heat tolerance. In addition, none of those genes reported in the previous studies have been experimentally verified to regulate heat tolerance in grapevines by transgenic methods. Therefore, it is essential to include heat-tolerant grapevine germplasms to acquire global insight into heat tolerance mechanisms and verify functionally of key heat response genes in grapevine.

Heat shock factors (HSFs) play critical, conserved roles in the plant transcriptional network that regulates thermo-responsive gene expression [21]. Based on the length of the flexible linker between DBD and HR-A/B regions and the number of amino acid residues in the HR-A/B regions, plant HSFs are classified into three classes: A, B, and C [22, 23]. Among class A HSFs, HSFA1s play critical roles in heat stress response and are regarded as indispensable regulators in the transcriptional network. In tomato and *Arabidopsis*, knockdown or knockout of *HSFA1* genes, downregulate many heat stress-responsive genes and induce heat stress-sensitive phenotypes [24–26]. HSFA1s directly regulate the expression of genes involved in heat stress response, including *DEHYDRATION-RESPONSIVE ELEMENT BINDING PROTEIN 2A* (*DREB2A*), *HSFA2*, *HSFA7a*, *HSFBs*, and *MULTIPROTEIN BRIDGING FACTOR 1C* (*MBF1C*) [25]. In addition to HSFA1s, other members of the class A family, such as HSFA2 and HSFA3, also play critical roles in plant response to heat stress. *HSFA2* is essential for heat stress response in plants. The *hsfa2*-knockout mutant exhibits high sensitivity to heat stress, together with reduced expression of many heat stress-inducible genes [27]. Knockout or knockdown of *HSFA3* also reduced the expression of HSP genes during heat stress [28–29]. Compared with the known activator function of several HSFAs in model plants, the roles of HSFBs are less well understood [30]. Moreover, compared to the model plants, much less research on HSFs has been carried out for fruit crops. *HSFAs* or *HSFBs* are significantly upregulated under heat stress in grape, apple, citrus, and strawberry [31–35]; however, only a few reports describe the roles of grape HSFs in heat stress with genetic evidence [36, 37].

In this study, we presented a high-resolution view of the transcriptional changes in heat tolerant (*Vitis davidii* ‘Tangwei’) and sensitive (*V. vinifera* ‘Jingxiu’) grapevines under varying degrees of high temperatures. The grape HSFB1 was identified and functionally characterized because it was differentially expressed only in ‘Tangwei’ and upregulated under heat stress. HSFB1 positively regulates heat tolerance in grapevine as a transcriptional repressor. Moreover, VdHSFB1 from ‘Tangwei’ and VvHSFB1 from ‘Jingxiu’, which differ only one amino acid, exhibit similar repression activity; however, the promoter variations of the two orthologues likely determine the difference of *HSFB1* gene expression in the two grape germplasms with contrasting thermotolerance, contributing to the difference in heat tolerance.

## Results

### Photosynthetic response of different grapevine germplasms to heat stress

**Figure 1:**
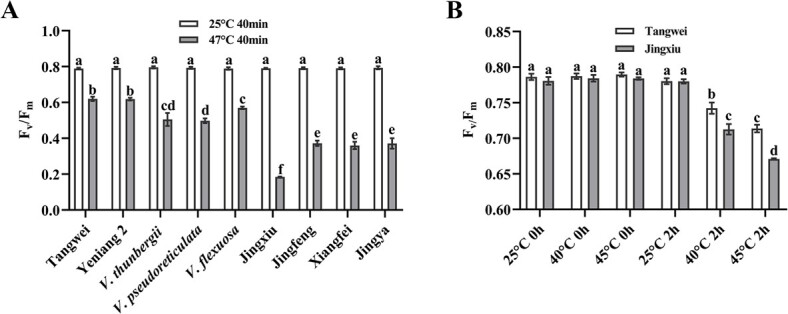
Photosynthetic efficiency of grapevine leaves under different temperatures. **A** Heat tolerance evaluation of nine grapevine germplasms based on the chlorophyll fluorescence parameters (*F_v_*/*F_m_*). *F_v_*/*F_m_* represents the potential maximum quantum yield of primary photochemistry of the detached mature leaves under the treatment of 25°C or 47°C for 40 min. *V. davidii* cv. ‘Tangwei’, *V. quinquangularis* cv. ‘Yeniang 2’, *V. thunbergii*, *V. pseudoreticulata* and *V. flexuosa* belong to wild species; ‘Jingxiu’, ‘Jingfeng’, and ‘Xiangfei’ belong to *V. vinifera*; ‘Jingya’ is a hybrid between *V. vinifera* and *V. labrusca*. **B** Photosynthetic efficiency changes of *V. davidii* ‘Tangwei’ and *V. vinifera* ‘Jingxiu’ grapevines under different temperatures. *F_v_*/*F_m_* represents the potential maximum quantum yield of primary photochemistry of the sixth leaves (from base to apex) of ‘Tangwei’ and ‘Jingxiu’ under the treatment of 25°C, 40°C, and 45°C for 0 h or 2 h. Data represent the mean ± SE of three biological replicates. Different letters indicate significant differences according to Duncan test (*P* < 0.05).

Photosynthesis is a sensitive physiological process affected by high temperature [38].The test of OJIP chlorophyll *a* fluorescence transient shows changes of photosystem II (PSII) electron transport chain. Heat injury under high temperature indirectly reflects plant heat tolerance and can be quickly evaluated by the parameter *F_v_*/*F_m_*, which represents the potential maximum quantum yield of primary photochemistry [39]. Based on our previous work that evaluated grapevine heat tolerance [39], we selected nine representative grapevine germplasms for further heat tolerance evaluation: *V. davidii* cultivar ‘Tangwei’, *V. quinquangularis* cultivar ‘Yeniang 2’, wild species *V. thunbergii*, *V. pseudoreticulata* and *V. flexuosa*; the *V. vinifera* cultivars ‘Jingxiu’, ‘Jingfeng’, and ‘Xiangfei’; and the *V. vinifera* × *V. labrusca* hybrid ‘Jingya’. Using the previously described method [39], the *F_v_/F_m_* values of detached grapevine leaves were measured after treatment at 25°C and 47°C for 40 min. The results showed that there was no significant difference in the *F_v_/F_m_* values among all grapevine germplasms at normal temperature treatment (Fig. 1A), However, the *F_v_/F_m_* values of the wild species were significantly higher than those of *V. vinifera* (Jingxiu, Jingfeng, and Xiangfei) and the *V. vinifera* × *V. labrusca* hybrid ‘Jingya’ after heat treatment (Fig. 1A), indicating that the wild species have relatively stronger heat tolerance than *V. vinifera* and its hybrid, consistent with previous reports [39]. ‘Tangwei’ and ‘Yeniang 2’ showed the highest heat tolerance, and ‘Jingxiu’ had the lowest (Fig. 1A). Therefore, we selected ‘Tangwei’ as a heat-tolerant germplasm and ‘Jingxiu’ as a heat-sensitive germplasm for comparative transcriptomic analysis. One-year-old ‘Tangwei’ and ‘Jingxiu’ grapevines were used to study the responses of grapevine germplasms with two heat treatments. There was no notable difference in the *F_v_/F_m_* values of ‘Tangwei’ and ‘Jingxiu’ leaves before different temperature treatments (Fig. 1B). After 40°C or 45°C treatment for 2 h, the *F_v_/F_m_* values of both ‘Tangwei’ and ‘Jingxiu’ declined significantly (*P* < 0.05) compared with the respective control groups (maintained at 25°C). Moreover, the *F_v_/F_m_* values decreased with the increase of treatment temperature. However, the *F_v_/F_m_* values of ‘Tangwei’ were significantly higher than those of ‘Jingxiu’ after exposure to 40°C or 45°C for 2 h (Fig. 1B). These results suggested that ‘Tangwei’ and ‘Jingxiu’ were injured by high temperatures, but the extent of heat injury was significantly lower in ‘Tangwei’ than ‘Jingxiu’.

### Transcriptome and DEG analysis of ‘Tangwei’ and ‘Jingxiu’ grapevines exposed to high temperatures

The libraries were sequenced by the Illumina HiSeq 2500 platform with three replication each sample. After quality controls, we aligned the clean reads to the *V. vinifera* reference genome sequence (12X, PN40024) (Table S1, see online supplementary material).

DEGs (|log2(fold change)| >1 and false discovery rate (FDR) <0.05) were defined as significantly upregulated or downregulated in one sample compared with another sample. The numbers of DEGs were determined for six comparisons: TW1 (Tangwei40°C vs Tangwei25°C), TW2 (Tangwei45°C vs Tangwei25°C), TW3 (Tangwei45°C vs Tangwei40°C), JX1 (Jingxiu40°C vs Jingxiu25°C), JX2 (Jingxiu45°C vs Jingxiu25°C), and JX3 (Jingxiu45°C vs Jingxiu40°C). The numbers of DEGs in each comparison were shown in Fig. S1A (see online supplementary material). Unique and shared DEGs for the six comparisons were indicated using Venn diagrams (Fig. S2B–E, see online supplementary material).

### Kyoto encyclopedia of genes and genomes (KEGG) enrichment analysis

KEGG annotations were used to identify DEG pathways, and 1400 DEGs from TW1 were assigned to 120 pathways (Additional file 1). For TW2, 2989 DEGs were assigned to 130 pathways (Additional file 1). The top four enriched pathways were metabolic pathway, biosynthesis of secondary metabolites, ribosome, and protein processing in endoplasmic reticulum in TW2 (Fig. S2C, see online supplementary material). For JX1, 1873 DEGs were assigned to 129 pathways, and for JX2, 1790 DEGs were assigned to 130 pathways (Additional file 1); the top four enriched pathways were metabolic pathway, biosynthesis of secondary metabolites, plant hormone signal transduction and protein processing in endoplasmic reticulum (Additional file 1). To validate the results of transcriptome analysis, qRT-PCR assays were performed on three DEGs (in Additional file 2) with gene-specific primers using RNA isolated from ‘Tangwei’ and ‘Jingxiu’ treated at 42°C for different times. The results showed that the significantly higher upregulation of *GOLS1* (galactinol synthase 1) and *GPX* (glutathione peroxidase) in ‘Tangwei’ compared to ‘Jingxiu’ (Fig. S3A and C, see online supplementary material), while the upregulation of *RS* (raffinose synthase) in ‘Jingxiu’ is higher than that in ‘Tangwei’ (Fig. S3B, see online supplementary material) These results were consistent with the transcriptome analysis.

### HSFs differentially expressed in ‘Tangwei’ and ‘Jingxiu’ in response to different high temperatures

**Figure 2:**
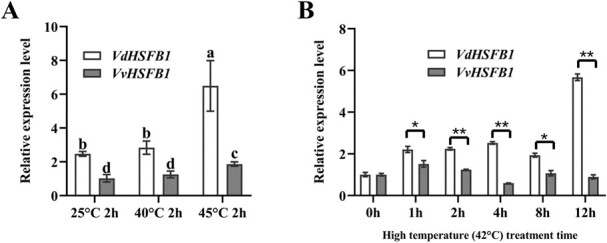
Expression levels of *HSFB1* in *V. davidii* ‘Tangwei’ and *V. vinifera* ‘Jingxiu’ grapevines under various heat treatments. **A** Analysis of the *HSFB1* expression in grapevine leaves of ‘Tangwei’ and ‘Jingxiu’ under 25°C, 40°C, and 45°C for 2 h. **B** Analysis of the *HSFB1* expression in grapevine leaves of ‘Tangwei’ and ‘Jingxiu’ under 42°C for 0, 1, 2, 4, 8, and 12 h. Different letters in **A** indicate significant differences among the treatments according to Duncan test (*P* < 0.05); in **B**, significant differences were determined using Student’s *t*-test: ^*^P < 0.05; ^**^P < 0.01.

Although there are a number of TFs in the above DEGs, the majority of the DEGs in protein processing in endoplasmic are HSFs, therefore the study focused HSFs. In grape, 19 putative *HSF* genes were identified [31, 32]. We further analyse HSFs respond to high temperature in grapevine. A total 11 *HSFs* (*HSFA1d*, *HSFA2*, *HSFA3*, *HSFA4a*, *HSFA6b*, *HSFA8*, *HSFB1*, *HSFB2a*, *HSFB2b*, *HSFB3*, and *HSFC1*) were identified in DEGs. The changes of these genes were shown in Fig. S4 (see online supplementary material) and Additional file 3. For example, VIT_204s0008g01110 encoding HSFA2 was markedly upregulated in both TW and JX comparisons. VIT_216s0100g00720 encoding HSFB2a and VIT_202s0025g04170 encoding HSFB2b showed greater upregulation in ‘Jingxiu’ than in ‘Tangwei’ under heat stress. The class A HSFs have been widely studied in plants [24–29], but function of class B and class C HSFs in heat tolerance are still unclear [30]. VIT_207s0031g00670 encoding HSFB1 was differentially expressed only in ‘Tangwei’ and was upregulated under heat stress (Additional file 3). In addition, *VpHSFB1a* (*HSFB1* in the study) significantly responds to heat stress in *V. pseudoreticulata* cultivar ‘Baihe-35-1’ [31]; and the expression of *HSF14* (*HSFB1* in the study) increases after the treatment at 38°C for 2 h followed by 47°C for 40 min compared to the treatment at 25°C for 2 h followed by 47°C for 40 min in ‘Tangwei’ [32]. To date, only the roles of HSFB2a and HSFB2b have been thoroughly studied in plants in HSFBs [40, 41]. Therefore, we chose HSFB1 for further study. The relative expression levels of *HSFB1* in ‘Tangwei’ and ‘Jingxiu’ were measured by using qRT-PCR. As expected, the expression of *HSFB1* was upregulated almost three-fold in ‘Tangwei’ at 45°C compared with 25°C, whereas its expression was upregulated only 1.5-fold in ‘Jingxiu’ under the same conditions (Fig. 2A).

The *HSFB1* expression level of ‘Tangwei’ was also significantly higher than that of ‘Jingxiu’ at both 25°C and 40°C (Fig. 2A). In addition, the upregulation folds of *HSFB1* expression in ‘Tangwei’ were higher than that in ‘Jingxiu’ under 42°C for a different treatment time (Fig. 2B). This result prompted us to explore if HSFB1 from ‘Tangwei’ and ‘Jingxiu’ influences the heat tolerance difference between these two grapevine germplasms.

### Nuclear localization and transcriptional repression activity of HSFB1

We cloned *VdHSFB1* and *VvHSFB1* from ‘Tangwei’ and ‘Jingxiu’, respectively. There are two SNPs (single nucleotide polymorphisms) difference between the coding regions of *VdHSFB1* and *VvHSFB1*, resulting in an amino acid difference (proline in VdHSFB1 and a leucine in VvHSFB1) (Figs S5 and S6A, see online supplementary material).

**Figure 3:**
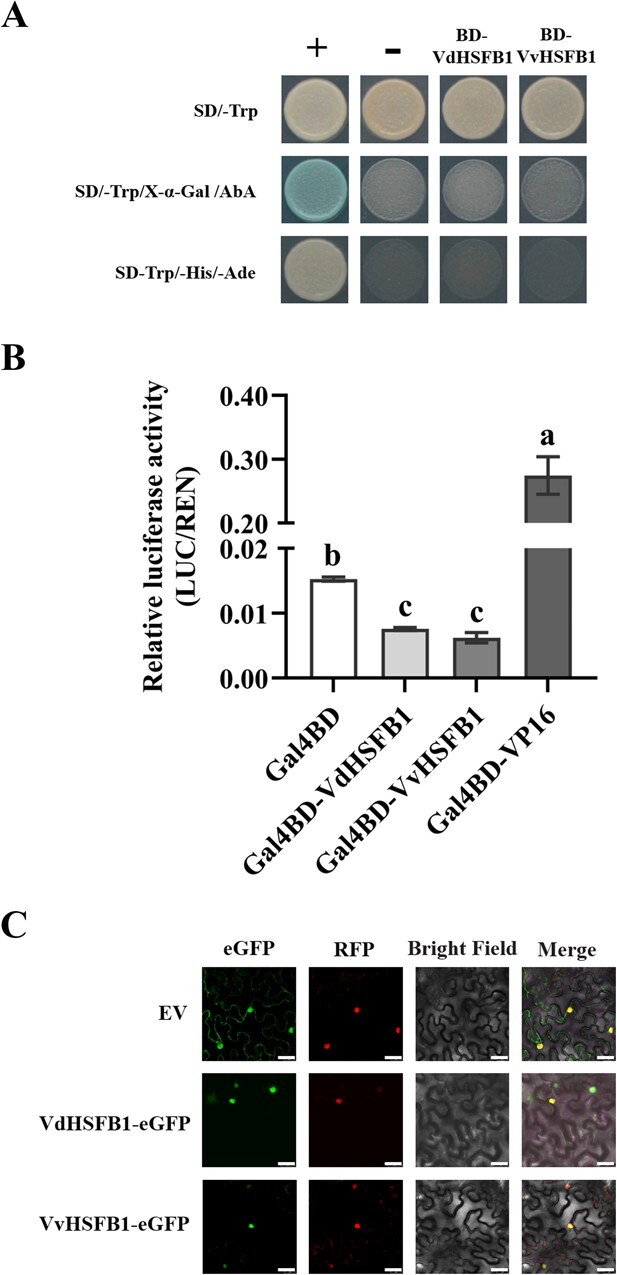
Transcriptional activity and subcellular localization of VdHSFB1 from *V. davidii* ‘Tangwei’ and VvHSFB1 from *V. vinifera* ‘Jingxiu’. **A** Analysis of VdHSFB1 and VvHSFB1 transcriptional activity in yeast. The coding sequence of *VdHSFB1* and *VvHSFB1* were fused with Gal4 binding domain (BD) of pGBDK7 vector, respectively. Constructed vectors were transformed into yeast strain Y2Hgold. Yeast cells expressing BD-VdHSFB1 and BD-VvHSFB1 were spotted on SD/−Trp, SD/−Trp/X-α-Gal/AbA, and SD-Trp/-His/−Ade selection media. Combinations of pGADT7-T with pGBKT7-Lam and pGBKT7-p53 were used as negative and positive controls, respectively. **B** Validation of VdHSFB1 and VvHSFB1 transcriptional activity in *Arabidopsis* protoplasts. The coding sequence of *VdHSFB1* and *VvHSFB1* were fused with Gal4 binding domain of Gal4BD vector, respectively. Constructed vectors were co-transformed into *Arabidopsis* protoplasts with LUC reporter vector and the REN vector. Gal4BD-VP16 and Gal4BD were used as positive and negative controls, respectively. Data represent the mean ± SE of three biological replicates. Different letters indicate significant differences according to Duncan test (*P* < 0.05). **C** Subcellular localization of VdHSFB1 and VvHSFB1 in tobacco leaves. VdHSFB1 and VvHSFB1 were fused with enhanced green fluorescent protein (eGFP), respectively. Constructed vectors were co-transformed with nuclear marker (H2B-mCherry) into tobacco leaves. eGFP fluorescence (green) and RFP fluorescence (red) were observed using a confocal microscope. Scale bars are 25 μm.

The respective proline and leucine are not located in any of the known functional domains, including DNA binding domain, HR-A/B domain, and B3 repression domain of HSFB1 (Fig. S6A, see online supplementary material). It has been suggested that the HSFBs have no transcriptional activation activity owing to the absence of an activation domain [42]. To explore the transcriptional activity of HSFB1, yeast and dual luciferase assays were performed. Yeast strain containing BD-VdHSFB1 or BD-VvHSFB1 did not turn blue on SD/−Trp/X-α-Gal/AbA medium or survive on SD-Trp/-His/−Ade selection medium, in contrast to the positive control (Fig. 3A), suggesting that neither proteins possess transcriptional activation activity. We then determined whether the two factors have transcriptional repression activity in a transactivation assay using *Arabidopsis* protoplasts. The relative luciferase activities of Gal4BD-VdHSFB1 and Gal4BD-VvHSFB1 were similar but significantly lower than that of Gal4BD (Fig. 3B), suggesting that VdHSFB1 and VvHSFB1 are repressors with similar transcriptional repression activities.

We then explored the possibility of the single amino acid change altering the subcellular localization of HSFB1. *VdHSFB1* and *VvHSFB1* were fused to *eGFP*, and the resulting fusion proteins VdHSFB1-eGFP and VvHSFB1-eGFP were separately co-transformed with P19 and the nuclear localization marker H2B-mCherry into tobacco leaves by agro-infiltration. After 3 days, the subcellular localization of VdHSFB1 and VvHSFB1 was determined by laser confocal microscopy. Both VdHSFB1 and VvHSFB1 co-localized in the nucleus with the nuclear localization marker (Fig. 3C), suggesting that the amino acid change did not alter HSFB1 subcellular localization. In addition, phylogenetic analysis suggested that both VdHSFB1 and VvHSFB1 exhibited the highest homology with *Citrus clementina* HSFB1 (CcHSFB1) (Fig. S6B, see online supplementary material).

### Increases of heat tolerance in heat sensitive grape plants by *HSFB1* overexpression

**Figure 4:**
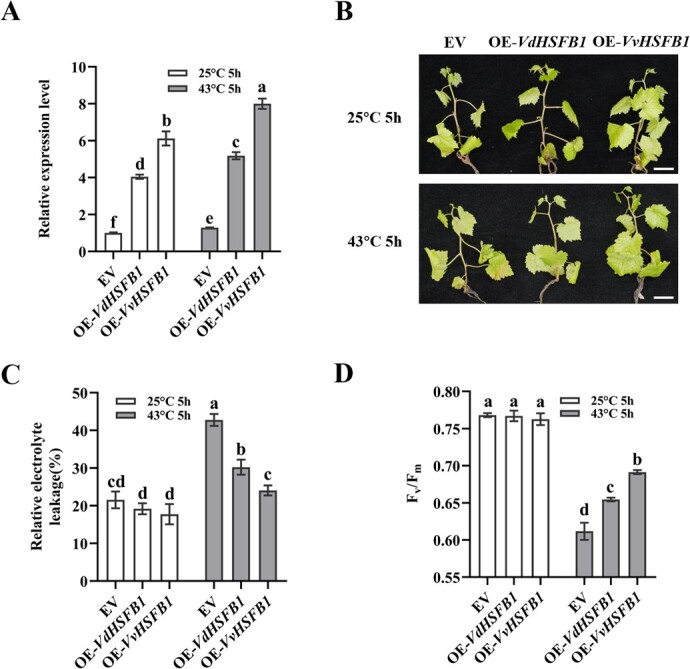
Transient overexpression of *VdHSFB1* and *VvHSFB1* improved heat tolerance in ‘Jingxiu’ plantlets. **A** Expression analysis of *HSFB1* in transient expression ‘Jingxiu’ plantlets of empty vector (EV), overexpressing *VdHSFB1* (OE-*VdHSFB1*), and overexpressing *VvHSFB1* (OE-*VvHSFB1*) using qRT-PCR. **B** The phenotype of empty vector (EV), overexpressing *VdHSFB1* (OE-*VdHSFB1*), and overexpressing *VvHSFB1* (OE-*VvHSFB1*) leaves under the treatment of 25°C or 43°C for 5 h. Scale bars are 2.5 cm. **C** The relative electrolyte leakage of empty vector (EV), overexpressing *VdHSFB1* (OE-*VdHSFB1*), and overexpressing *VvHSFB1* (OE-*VvHSFB1*) leaves under the treatment of 25°C or 43°C for 5 h. **D** Heat tolerance evaluation of empty vector (EV), overexpressing *VdHSFB1* (OE-*VdHSFB1*) and overexpressing *VvHSFB1* (OE-*VvHSFB1*) plantlets based on the chlorophyll fluorescence parameters (*F_v_*/*F_m_*). The *F_v_*/*F_m_* values represent the potential maximum quantum yield of primary photochemistry of the leaves under the treatment of 25°C and 43°C for 5 h. Data represent the mean ± SE of three biological replicates. Different letters indicate significant differences according to Duncan test (*P* < 0.05).

Due to the difficulty of obtaining the stable transgenic grape plants, *HSFB1* was transiently overexpressed in ‘Jingxiu’ plantlets to determine its function in heat tolerance. The coding sequence of *VdHSFB1* and *VvHSFB1* were cloned into the overexpression vector pCAMBIA-2300 to create OE*-VdHSFB1* and OE*-VvHSFB1*. The overexpression vectors and the empty vector (EV) were transformed into *Agrobacterium tumefaciens* EHA105. The transformed Agrobacteria were infiltrated into ‘Jingxiu’ leaves. After incubation for 3 days, the leaves of EV, OE-*VdHSFB1*, and OE-*VvHSFB1* plantlets were sampled for qRT-PCR to measure *HSFB1* expression. The relative expression levels of *HSFB1* in OE-*VdHSFB1* and OE-*VvHSFB1* were 3- and 6-fold more than that in EV, respectively (Fig. 4A).

Then EV, OE-*VdHSFB1*, and OE-*VvHSFB1* plantlets were placed in an incubator at 43°C. After 5 h, the infiltrated leaves of EV became exsiccated compared to those of OE-*VdHSFB1* and OE-*VvHSFB1* plantlets, which remain normal (Fig. 4B). In addition, the relative electrolyte leakage of EV plantlets was significantly higher than that of OE-*VdHSFB1* or OE-*VvHSFB1* plantlets under high temperature (Fig. 4C), Correspondingly, the *F_v_*/*F_m_* values of EV were significantly lower than those of OE-*VdHSFB1* or OE-*VvHSFB1* leaves (Fig. 4D). Taken together, these results indicated that overexpression of *HSFB1* enhances heat tolerance in grape plantlets.

### Decrease of heat tolerance of grape plants by suppression of *HSFB1*

Due to the rooting difficulty of ‘Tangwei’, we were unable to generate transformants of ‘Tangwei’. We thus chose ‘Yeniang 2’ (*V. quinquangularis*), exhibiting similar heat tolerance as ‘Tangwei’ (Fig. 1A), for generation of tissue culture plants. The coding sequences of *HSFB1* are identical in ‘Yeniang 2’ and ‘Tangwei’ (Fig. S7, see online supplementary material).

**Figure 5:**
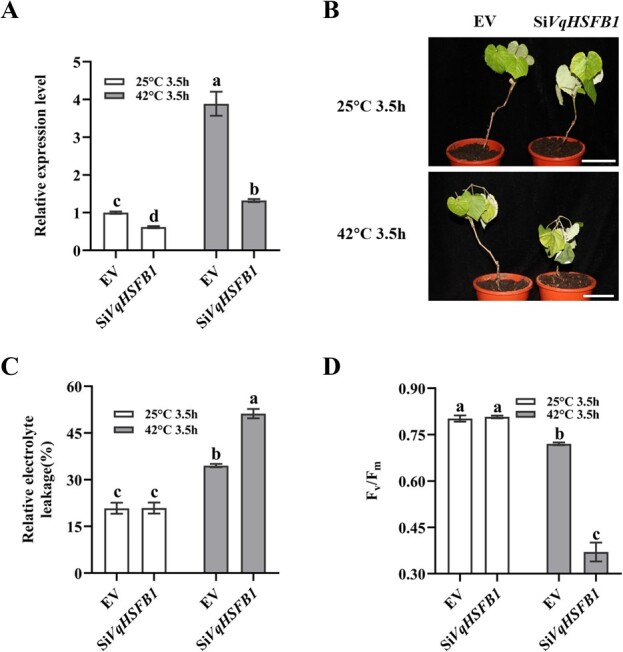
Transient suppression of *VqHSFB1* enhanced heat sensitivity in ‘Yeniang 2’ young plants. **A** Expression analysis of *HSFB1* in transient expression ‘Yeniang 2’ young plants of empty vector (EV), RNA interference of *VqHSFB1* (Si*VqHSFB1*) using qRT-PCR. **B** The phenotype of empty vector (EV), RNA interference of *VqHSFB1* (Si*VqHSFB1*) plants under the treatment of 25°C or 42°C for 3.5 h. Scale bars are 5 cm. **C** The relative electrolyte leakage of empty vector (EV), RNA interference of *VqHSFB1* (Si*VqHSFB1*) plants under the treatment of 25°C or 42°C for 3.5 h. **D** Heat tolerance evaluation of empty vector (EV), RNA interference of *VqHSFB1* (Si*VqHSFB1*) plants based on the chlorophyll fluorescence parameters (*F_v_*/*F_m_*). The *F_v_*/*F_m_* values represent the potential maximum quantum yield of primary photochemistry of the leaves under the treatment of 25°C and 42°C for 3.5 h. Data represent the mean ± SE of three biological replicates. Different letters indicate significant differences according to Duncan test (*P* < 0.05).

For RNA interference, a 271-bp fragment of *VqHSFB1* was inserted into the vector pFGC5941. The resultant Si*VqHSFB1* and EV were mobilized into *A. tumefaciens* EHA105. The transformed Agrobacteria were infiltrated into the leaves of one-year-old ‘Yeniang 2’ plants which had germinated for 8 weeks. After 5 days, the leaves of EV and Si*VqHSFB1* plants were sampled to determine the *VqHSFB1* expression using qRT-PCR. The relative expression level of *VqHSFB1* in Si*VqHSFB1* plants was significantly lower than that in EV (Fig. 5A).

EV and Si*VqHSFB1* plants were placed in an incubator with light at 42°C. After 3.5 h, the leaves of Si*VqHSFB1* plants became significantly more wilted compared with EV or Si*VqHSFB1* plants at 25°C (Fig. 5B). Moreover, the relative electrolyte leakage of Si*VqHSFB1* was notably higher than that of EV plants after heat treatment (Fig. 5C). In addition, the *F_v_*/*F_m_* values of EV leaves were approximately 2-fold higher than that of Si*VqHSFB1* leaves under high temperature (Fig. 5D). The results indicated that downregulation of *HSFB1* expression decreases heat tolerance of grape plants.

### Enhancement of heat tolerance of grape suspension cells stably overexpressing *HSFB1*

The one amino acid difference between VdHSFB1 and VvHSFB1 does not alter the basic characteristics and functions of the two proteins (Figs 3–5; Fig. S6, see online supplementary material), *VdHSFB1* was thus chosen to represent *HSFB1* for further study. To explore whether *HSFB1* confers heat tolerance in a stable transgenic system, *VdHSFB1* was overexpressed in suspension cells of ‘41B’ (*V. vinifera* ‘Chasselas’ × *V. berlandieri*). ‘41B’ is identical to *VvHSFB1* (Fig. S8, see online supplementary material).

**Figure 6:**
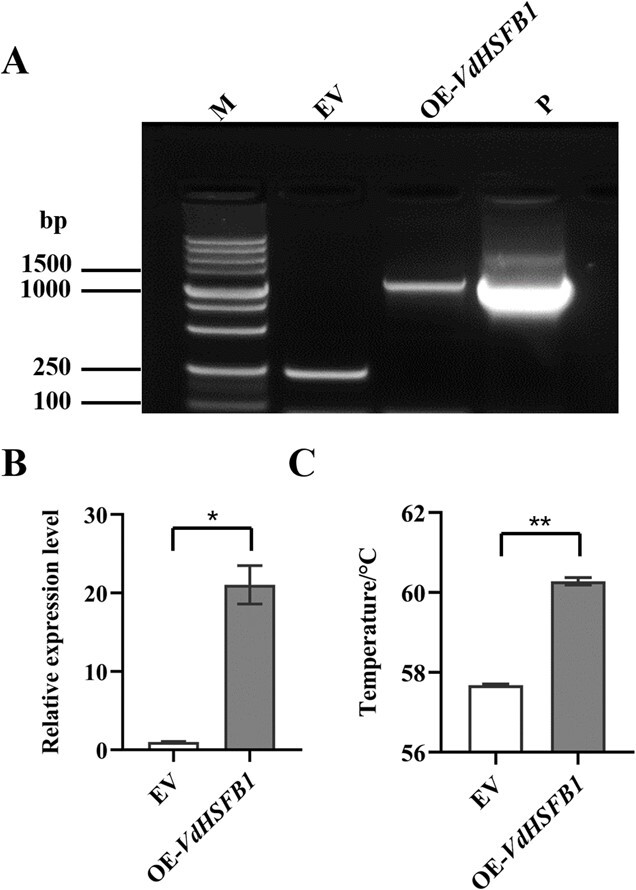
Stable overexpression of *VdHSFB1* improved heat tolerance in grape suspension cells. **A** Detection of transgenic suspension cells of ‘41B’ (*V. vinifera* ‘Chasselas’ × *V. berlandieri*) by PCR. The cells transformed with empty vector (EV) was as control. M indicates the marker; P indicates the plasmid of OE-*VdHSFB1*. **B** Expression analysis of *HSFB1* in transgenic grape suspension cells of empty vector (EV), overexpressing *VdHSFB1* (OE-*VdHSFB1*) using qRT-PCR. **C** The T_COND_ values of grape suspension cells of EV and OE-*VdHSFB1*. The electrical conductivity of the transgenic suspension cells was measured during continuously heating, which abruptly increases at a certain temperature which is called as T_COND_. The T_COND_ reflects the heat tolerance of organisms or cells. Data are means (± SE) from three independent biological replicates. Significant differences were determined using Student’s *t*-test: ^*^P < 0.05; ^**^P < 0.01.

The suspension cells were transformed as described previously [43]. Transgenic status and *VdHSFB1* expression were verified by PCR and qRT-PCR assays (Fig. 6A and B).

The relative expression of *VdHSFB1* in OE-*VdHSFB1* was 20-fold higher than that in EV (Fig. 6B). The heat tolerance of OE-*VdHSFB1* was then assessed by measuring the critical electrical conductivity temperature (T_COND_) as previously described [44]. The T_COND_ of OE-*VdHSFB1* was approximately 2°C higher than that of EV (Fig. 6C), suggesting that *VdHSFB1* confers grape cell heat tolerance.

### Cloning and sequence analysis of the *HSFB1* promoters

The same transcriptional repression activity between VdHSFB1 and VvHSFB1 prompted us to speculate that the differential expression of *VdHSFB1* and *VvHSFB1* is due to a difference in promoter. We thus cloned the promoters of *VdHSFB1* and *VvHSFB1.* Sequence alignment revealed that there were 27 SNPs and 11 Indels in the promoters of *VdHSFB1* and *VvHSFB1* (Fig. S9, see online supplementary material).

To investigate whether the SNPs and Indels in the *HSFB1* promoters alter the *cis*-elements, the Plant Cis-Acting Regulatory Element (Plant CARE) database was used to analyse the *VdHSFB1* and *VvHSFB1* promoters [45]. The types of *cis*-elements in the *VdHSFB1* and *VvHSFB1* promoters remain the same; however, the promoter sequence variations resulted in more ARE, AT~TATA-box, MYB, and TATA-box *cis*-elements in the *VdHSFB1* promoter than the *VvHSFB1* promoter (S2, see online supplementary material).

### Analysis of the *HSFB1* promoter activity in grape plantlets and *Arabidopsis* protoplasts

**Figure 7:**
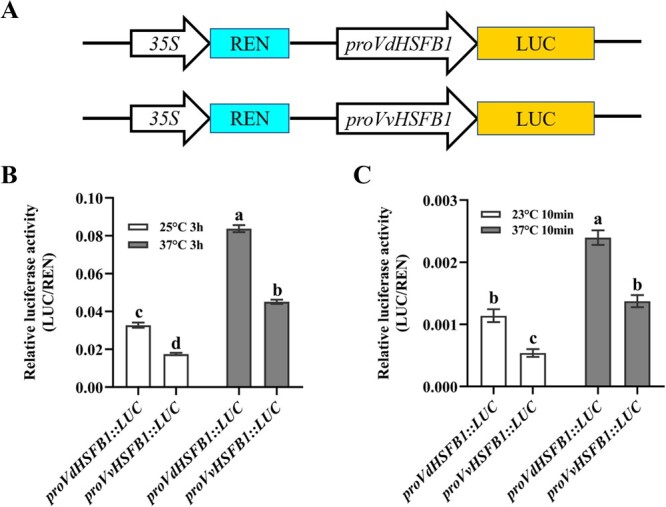
Promoter activity of *VdHSFB1* and *VvHSFB1* in grape plantlets and *Arabidopsis* protoplasts under normal and high temperature treatments. **A** Constructs used in the dual-luciferase reporter assay. The promoter of *VdHSFB1* and *VvHSFB1* were fused with pGreenII-0800-LUC vector. **B** Analysis of the promoter activity of *VdHSFB1* from ‘Tangwei’ and *VvHSFB1* from ‘Jingxiu’ in ‘Jingxiu’ plantlet leaves under 25°C and 37°C for 3 h. Constructed vectors were transformed into ‘Jingxiu’ plantlets, respectively. **C** Analysis of the promoter activity of *VdHSFB1* from ‘Tangwei’ and *VvHSFB1* from ‘Jingxiu’ in *Arabidopsis* protoplasts under 23°C and 37°C for 10 min. Constructed vectors were transformed into the prepared *Arabidopsis* protoplasts, respectively. The ratio of LUC/REN represents the relative luciferase activity. Data represent the mean ± SE of three biological replicates. Different letters indicate significant differences among the treatments according to Duncan test (*P* < 0.05).

The heat tolerance of transgenic plantlets was found to be associated with the expression level of *HSFB1* (Fig. 4). We next tested the promoter activities of *VdHSFB1* and *VvHSFB1*. *Cis*-elements analysis showed that there were 10 more TATA-boxes in the *VdHSFB1* promoter than the *VvHSFB1* promoter, and TATA-box is associated with promoter strength [46]. We measured the promoter activities of *VdHSFB1* and *VvHSFB1* in grape plantlets and *Arabidopsis* protoplasts. The promoters of *VdHSFB1* and *VvHSFB1* were individual in fusion with the luciferase reporter gene (LUC) in the pGreenII-0800-LUC vector (Fig. 7A). The resultant vectors, *proVdHSFB1::LUC* and *proVvHSFB1::LUC,* were mobilized into *A. tumefaciens* GV3101 (pSoup), and then transformed into the ‘Jingxiu’ plantlets. After incubation for 3 days, the leaves of *proVdHSFB1::LUC* and *proVvHSFB1::LUC* plantlets were sampled to measure the luciferase activity. The relative luciferase activity of *proVdHSFB1* was significantly higher than that of *proVvHSFB1* under both normal and high temperatures compared to that of *VvHSFB1* in grape plantlets (Fig. 7B). In addition, the promoter activities of both *VdHSFB1* and *VvHSFB1* were higher when assayed at 37°C compared to 25°C (Fig. 7B). We also detected the promoter activity of *VdHSFB1* and *VvHSFB1* in *Arabidopsis* protoplasts. The plasmids of *proVdHSFB1::LUC* and *proVvHSFB1::LUC* were transformed into prepared *Arabidopsis* protoplasts, respectively. After incubation at 23°C for 16 h, the protoplasts transfected with *proVdHSFB1::LUC* and *proVvHSFB1::LUC* were placed in temperature-controlled (23°C and 37°C) water baths for 10 min before measurement of LUC and REN activities. The relative luciferase activity of *proVdHSFB1* was significantly higher than that of *proVvHSFB1* when assayed at 23°C, and at elevated temperature (37°C) the luciferase activities driven by both promoters increased, compared to those at 23°C, while the *proVdHSFB1* activity remained notably higher than that of *proVvHSFB1* (Fig. 7C). These results showed that the promoter activity of *VdHSFB1* is significantly higher than that of *VvHSFB1*.

## Discussion

In the past decades, climate change significantly influenced grape production. Worldwide viticulture will encounter a serious threat in the near future if annual temperatures continue to rise [47]. Most global cultivars derived from mild climates may be unable to withstand the extreme heat stress [48, 49]. Grapevine germplasms from warmer regions may therefore be important genetic resources. Exploring the molecular mechanisms underlying heat tolerance of these germplasms is particularly important for breeding new heat-tolerant cultivars, thereby promoting the sustainability of grape cultivation and the wine industry [50, 51]. Here, we evaluated the heat tolerance of nine representative germplasms, and found that the heat tolerance between *V. davidii* ‘Tangwei’ or *V. quinquangularis* ‘Yeniang 2’ and *V. vinifera* ‘Jingxiu’ was the most different (Fig. 1A). The strong heat tolerance of *V. davidii* and *V. quinquangularis* may be closely associated with their distribution in southern China and their long history of growth in hot and humid conditions. Although showing many similarities in heat response as other crops, the perennial grapes possess distinct characteristics [52]. We used *V. davidii* ‘Tangwei’ (heat tolerant) and *V. vinifera* ‘Jingxiu’ (heat sensitive) as two contrasting germplasms to investigate the specific factors in the regulatory networks that control grapevine response to high temperature.

### More DEGs are found in heat-tolerant grapevine germplasms in response to heat stress

Transcriptomics is useful to understand how plants respond to abiotic stresses and to characterize genes involved in these responses. Together with the physiological data, transcriptional profiling aided us to dissect the molecular programming that distinguishes the heat stress responses between the heat tolerant and sensitive germplasms. The numbers of DEGs in both ‘Tangwei’ and ‘Jingxiu’ increased with increasing treatment temperature; however, the DEG numbers in ‘Tangwei’ (757) were significantly greater than in ‘Jingxiu’ (304) (Fig. S1, see online supplementary material), suggesting that the heat-tolerant germplasm activates more genes under higher temperature and can better respond to heat stress compared with the heat-sensitive germplasm. Moreover, there were far more unique DEGs in ‘Tangwei’ than ‘Jingxiu’ when comparing each germplasm at elevated temperature to normal temperature, i.e. 4337 and 4122 DEGs in TW2 and TW3, respectively, versus 2437 and 748 in JX2 and JX3, respectively (Fig. S1D and E, see online supplementary material), suggesting that more genes are affected in ‘Tangwei’ than in ‘Jingxiu’. The heat-tolerant germplasms seem to evolve more larger regulatory networks to adapt heat stress.

### HSFB1 is a key factor determining the difference between heat tolerant and sensitive grapevines

HSFs are considered to be one of the most important TF families for plant heat stress response [53]. Here, 11 grape HSFs were also identified as DEGs in response to heat treatments. Similar to the previous studies, the expression of VIT_204s0008g01110 (*HSFA2*) showed marked upregulation in the TW1, TW2, JX1, and JX2 comparisons (Additional file 3), indicating that *HSFA2* may contribute to enhancing grapevine heat tolerance. Interestingly, HSFB1 from the HSFB family was identified as a DEG in the TW2 and TW3 comparisons but not in any JX comparisons (Additional file 3), which was confirmed by qRT-PCR assays (Fig. 3A). In addition, the upregulation of *HSFB1* in ‘Tangwei’ was higher than that in ‘Jingxiu’ under 42°C for a different treatment time (Fig. 2B). The above results suggest that HSFB1 may determine the difference in heat tolerance between heat tolerant and sensitive grapevines.

### HSFB1 positively regulates heat tolerance as a transcriptional repressor in grapevine

HSFAs are commonly transcriptional activators due to the acidic AHA domains with activator potential [23]. Some HSFBs possess transcriptional repression activities due to the presence of B3 repression domains [42, 54]. Here, we showed that the HSFB, HSFB1, possesses a B3 repression domain and represses transcription in an *Arabidopsis* protoplast-based assay (Fig. 3B; Fig. S6A, see online supplementary material). The VdHSFB1 and VvHSFB1 proteins only differ in one amino acid which does not seem to affect transcriptional repression activity (Fig. 3B). Some HSFBs possess transcriptional repression or activation activity in plants. In tomato, HSFB1 not only functions as a repressor to repress the expression of HS-inducible genes but also as a co-activator of HSFA1a and other non-HSF TFs [55]. In *Arabidopsis*, HSFB2b is a transcriptional repressor of several HSP genes [41]. However, chickpea HSFB2 has weaker transcription activation activity [56]. *HSFBs* expression are often induced by heat stress; however, their roles in plant heat tolerance can be postive or negative. For example, *TaHSF3,* a member of HSFBs with transcriptional repression activity, enhances heat tolerance of *Arabidopsis* [57]. In contrast, the *Arabidopsis* HSFB1 and HSFB2 is a transcriptional repressor [54], but the *hsfb1hsfb2b* mutants exhibit higher basal thermotolerance than the wild type plant [41]. We overexpressed and RNAi *VdHSFB1* and *VvHSFB1* in grapevines. We found that transient overexpression of *VdHSFB1* and *VvHSFB1* enhanced heat tolerance in ‘Jingxiu’ (Fig. 4), and RNAi of *HSFB1* decreased heat tolerance in a heat tolerant germplasm (Fig. 5). Moreover, overexpression of *VdHSFB1* significantly enhanced the heat tolerance of OE-*VdHSFB1* compared with EV suspension cells (Fig. 6). Furthermore, *HSFB1* overexpression was positively correlated to heat tolerance (Fig. 4). These results suggest that the grape *HSFB1* positively regulates heat tolerance in grapevine.

### Promoter variations between *VdHSFB1* and *VvHSFB1* contribute to heat tolerance difference between ‘Tangwei’ and ‘Jingxiu’

The induction of HSFBs in plants under abiotic stress have been reported. The expression of *OsHSF2b* is highly induced by heat, salt, and polyethylene glycol (PEG) treatments in rice [58]. *TaHSF3* is significantly upregulated under heat and cold stress in wheat seedlings [57]. The expression of *CarHSFB2* is upregulated under the heat, salt, and drought stress [56]. Here, *VdHSFB1* and *VvHSFB1* was induced by high temperatures. Moreover, the extent of upregulation of *VdHSFB1* was notably higher than that of *VvHSFB1* under different high temperatures (Fig. 2A) and under 42°C for different treatment time (Fig. 2B). These results and the genetic evidence (Figs 4–6) indicated the *HSFB1* expression level correlates positively with grape heat tolerance. In rice, the promoter sequence variations of *CTB4a* confer varying degrees of cold tolerance [59]. Similarly, the sequence variations in the *SLG1* promoter increase the thiolated tRNA level, thus enhancing heat tolerance of *indica* rice varieties [60]. Therefore, we speculated that the expression difference between *VdHSFB1* and *VvHSFB1* is associated with difference in the promoter activity. In this study, the transient expression assays using plantlets and *Arabidopsis* protoplasts showed that the promoter activity of *VdHSFB1* is notably higher than that of *VvHSFB1* under both normal and high temperatures (Fig. 7). In addition, we found more TATA-box and AT~TATA-box *cis*-elements in *VdHSFB1* promoter than the *VvHSFB1* promoter (Table S2, see online supplementary material). TATA-box has been shown to enhance transcription efficiency [61]. Therefore, promoter variations between *VdHSFB1* and *VvHSFB1* influence heat difference of grapevines. Further study will be needed to pinpoint the role of each *cis*-element in enhancement of the promoter strength.

## Conclusions

This study provides abundant transcriptomic data on the response of heat-tolerant (‘Tangwei’) and heat-sensitive (‘Jingxiu’) grapevines responding to different high temperatures. The significantly enriched DEGs in the heat-tolerant germplasm provide many candidate genes for future studies of the molecular mechanisms governing heat-tolerance in grapevines. In addition, we demonstrated that the grape HSFB1 is a critical factor involved in thermotolerance. VdHSFB1 and VvHSFB1 from the two contrasting germplasms possess similar transcriptional repression activity although the two proteins only differ in one amino acid. In addition, *HSFB1* expression levels are markedly different in ‘Tangwei’ and ‘Jingxiu’. We propose that the difference in the expression levels is due to the promoter strengths, which can be explained by promoter sequence variation that results in change of the number of *cis*-elements. Promoter variations between VdHSFB1 and VvHSFB1 contribute to heat tolerance difference between the two grape germplasms with contrasting thermotolerance.

## Materials and methods

### Plant materials and heat treatments

Nine grapevine germplasm resources (*V. davidii* ‘Tangwei’, *V. quinquangularis* ‘Yeniang 2’, *V. thunbergii*, *V. pseudoreticulata*, *V. flexuosa*, *V. vinifera* ‘Jingxiu’, ‘Jingfeng’, and ‘Xiangfei’ and *V. vinifera* × *V. labrusca* ‘Jingya’) were planted at grapevine germplasm resource bank of the Institute of Botany, Chinese Academy of Sciences, Beijing, China. The heat tolerance of these grapevines was assessed using a previously described protocol [39].

One-year-old ‘Tangwei’ and ‘Jingxiu’ grapevines were cultivated in pots. The culture conditions were previously reported [17]. When the sixth leaves (measured from the base) of the grape plants had matured (about 30 days old), all grapevines were transferred to a controlled-environment room with similar culture conditions as previously reported [17]. These grape plants were divided into six groups (three temperature treatments × two germplasms) and adapted for 2 d. Then the grapevines were exposed to 25°C, 40°C, or 45°C for 2 h. The *F_v_*/*F_m_* values of the sixth leaves were recorded with a Handy Plant Efficiency Analyzer made by Hansatech (Norfolk, UK) at the end of the treatment period. These leaves were wrapped with tin foil and then put in liquid nitrogen. Three grapevines were used for each biological replicate, and three replicates were used for both treatments (40°C and 45°C) and controls (25°C). In addition, as for ‘Tangwei’ and ‘Jingxiu’ grapevines treated under 42°C for different times, when the sixth leaves (measured from the base) of the grape plants had matured (about 30 days old), these grapevines were divided into 12 groups (six time treatments × two germplasms) and adapted for 2 d. Then the grapevines were exposed to 42°C for 0, 1, 2, 4, 8, and 12 h, respectively. The sixth leaves (measured from the base) of the grape plants were wrapped with tin foil and then put in liquid nitrogen. Three plants were used for each biological replicate, and three replicates were used for treatments (42°C) and controls (25°C).

*Arabidopsis thaliana* (Col-0) and *Nicotiana benthamiana* in potting nutrient soil were cultured under 23°C with a 14-h photoperiod.

41B embryogenic calli was initiated as described previously [62]. The culture and sub-cultured methods were previously described [63].

‘Jingxiu’ plantlets were grown on half-strength Murashige and Skoog (½MS, pH 5.8, Phyto-Tech) solid medium under 25°C with a 14-h photoperiod. ‘Yeniang 2’ plants were grown in potting nutrient soil under 25°C with a 14-h photoperiod.

### RNA sequencing analysis

RNA sequencing was performed at Biomarker Technologies Corporation (Beijing, China). Data processing and analysis were conducted as previously described [64]. In brief, low-quality reads, adapter sequences and reads with ambiguous bases were removed by Trimmomatic v0.36 [65]. The following parameters were used to obtain high-quality reads: LEADING: 3, TRAILING: 3, SLIDINGWINDOW: 4:15, and MINLEN: 90. All clean reads were mapped to the *V. vinifera* reference genome (version 12×) using TopHat (v2.1.1) [66, 67]and allowed no more than 2 bp of mismatch.

Gene annotation, expression quantification, and DE analysis were performed using Cufflinks software (v2.2.1) [68]. Gene expression normalization of every sample was performed using DESeq2, and DEGs were identified based on fold change >2 and false discovery rate (FDR) <0.05.

### KEGG pathway enrichment analysis

The statistical enrichment of DEGs in KEGG pathways used KOBAS software [69].

### Quantitative reverse transcription-PCR analysis

qRT-PCR was used to detect gene expression according to the method previously described [17]. The primers for qRT-PCR are shown in Table S3 (see online supplementary material).

### Generation of transgenic plants and heat treatments

The coding sequence of *VdHSFB1* and *VvHSFB1* were cloned from leaves of ‘Tangwei’ and ‘Jingxiu’, respectively. Then *VdHSFB1* and *VvHSFB1* were subcloned into the overexpression vector pCAMBIA2300 to generate OE-*VdHSFB1* and OE-*VvHSFB1*. Then Empty Vector (EV), OE-*VdHSFB1* and OE-*VvHSFB1* were mobilized into *A. tumefaciens* EHA105 (ZOMANBIO Company, ZC142), respectively. Six-week-old ‘Jingxiu’ plantlets were used for transient overexpression assays. The plantlets were infiltrated by EV, OE-*VdHSFB1*, and OE-*VvHSFB1* under vacuum (−90 kPa) for 20 min, respectively. Then, ddH_2_O was used to wash these plantlets and blotting paper was used to dry these plantlets, and the roots were inserted into ½MS medium and put in the greenhouse under 25°C with a 14-h photoperiod. After 3 days, these plantlets were sampled and phenotype evaluated. Plantlets in bottles were treated without opening the lids at the following temperatures: (i) the control plantlets were maintained at 25°C for 5 h; and (ii) the treatment plantlets were treated at 43°C for 5 h. Meanwhile, The *F_v_*/*F_m_* values of the third leaves from the root were recorded with a Handy Plant Efficiency Analyzer made by Hansatech (Norfolk, UK). The primers for constructing OE-*VdHSFB1* and OE-*VvHSFB1* are in Table S3 (see online supplementary material).

To knock down the expression of *HSFB1* in ‘Yeniang 2’, a 271-bp fragment (587 to 858 bp) of *VqHSFB1* coding sequence was fused with pFGC5941 in sense and antisense orientation using a previously described method [70, 71]. The binary construct named Si*VqHSFB1* and Empty Vector (EV) were mobilized into *A. tumefaciens* EHA105 (ZOMANBIO Company, ZC142). One-year-old ‘Yeniang 2’ plants that had germinated for 8 weeks were used for transient interference assays. These plants were vacuum (−90 kPa) infiltrated by EV and Si*VqHSFB1* for 20 min, respectively. Then, ddH_2_O was used to wash these plants and blotting paper was used to dry these plants, and the plants were cultured in a small pot containing nutrient soil in a growth room under 25°C with a 14-h photoperiod. After 5 days, these plants were used for phenotype evaluation. The plants were treated by different temperatures: (i) the control plants were maintained at 25°C for 3.5 h; and (ii) the treatment plants were treated at 42°C for 3.5 h. Meanwhile, the *F_v_*/*F_m_* values of the fifth leaves from the root were recorded with a Handy Plant Efficiency Analyzer made by Hansatech (Norfolk, UK). The primers for constructing Si*VqHSFB1* are in Table S3 (see online supplementary material).

In addition, *VdHSFB1* was then transformed into suspension cells of ‘41B’ (*V. vinifera* ‘Chasselas’ × *V. berlandieri*). Transgenic suspension cells were generated using a previously described method [43]. After antibiotic screening, transgenic suspension cells were obtained. The primers for cloning and transformation are in Table S3 (see online supplementary material). Appropriate 41B suspension cells were put in a water bath, and water at room temperature was heated from 25°C to 65°C. The electrical conductivity of the transgenic suspension cells was determined using PlanTherm PT100 made by Photon Systems (Brno, Czech Republic)during continuous heating. It increased abruptly at a specific temperature called T_COND_, which reflects the heat tolerance of organisms or cells [44].

### Transient luciferase (LUC) expression assays

The 2518-bp (*VdHSFB1*) and 2541-bp (*VvHSFB1*) promoter sequences were cloned from DNA extracted from the leaves of ‘Tangwei’ and ‘Jingxiu’, respectively. The *VdHSFB1* and *VvHSFB1* promoters were subcloned into the pGreenII-0800-LUC vector. Then *proVdHSFB1::LUC* and *proVvHSFB1::LUC* were mobilized into *A. tumefaciens* GV3101 (pSoup) (ZOMANBIO Company, ZC142), respectively. Six-week-old ‘Jingxiu’ plantlets were used for transient luciferase (LUC) expression assays. The plantlets were infiltrated by EV, OE-*VdHSFB1*, and OE-*VvHSFB1* under vacuum (−90 kPa) for 20 min, respectively. Then, ddH_2_O was used to wash these plantlets and blotting paper was used to dry these plantlets, and the roots were inserted into ½MS medium and put in the greenhouse under 25°C with a 14-h photoperiod. After 3 days, the leaves of these plantlets were sampled after temperature treatments. Plantlets in bottles were treated without opening the lids at the following temperatures: (i) the control plantlets were maintained at 25°C for 3 h; and (ii) the treatment plantlets were treated at 37°C for 3 h. Then the LUC activity and REN activity of these leaves were determined by using the Dual-Luciferase® Reporter Assay System (E1910, Promega). 400 μL protein lysis buffer was added into the 0.05 g leaves of grape plantlets for total protein extraction. Then the activities of LUC and REN were measured by using GloMax 20/20 luminometer made by Promega (Wisconsin, Madison, USA). The ratio of LUC/REN stands for the promoter activity of *VdHSFB1* and *VvHSFB1*, respectively.

The plasmid of *proVdHSFB1::LUC* and *proVvHSFB1::LUC* were transformed into prepared *Arabidopsis* protoplasts. After incubation at 23°C for 16 h, the protoplasts transfected with *VdHSFB1* and *VvHSFB1* were placed in temperature-controlled (23°C and 37°C) water baths for 10 min. The LUC activity and REN activity of these *Arabidopsis* protoplasts were determined by using the Dual-Luciferase® Reporter Assay System (E1910, Promega). 100 μL protein lysis buffer was added into the *Arabidopsis* protoplasts for total protein extraction and then the activities of LUC and REN were measured by using GloMax 20/20 luminometer (made by Promega (Wisconsin, Madison, USA).

The coding sequences of *VdHSFB1* and *VvHSFB1* were fused with the 35S-GAL4 vector, and Gal4BD-VdHSFB1 and Gal4BD-VvHSFB1 were transformed into *Arabidopsis* protoplasts with LUC and REN. After incubation at 23°C for 16 h, the LUC activity and REN activity of these *Arabidopsis* protoplasts were determined by using the Dual-Luciferase® Reporter Assay System (E1910, Promega). The activities of LUC and REN were determined as described above. 35S-GAL4 (Gal4BD) and 35S-GAL4-VP16 (Gal4BD-VP16) were the negative and positive and control, respectively. The primers for luciferase expression assays are shown in Table S3 (see online supplementary material).

### Yeast assays

*VdHSFB1* and *VvHSFB1* coding sequences were individually fused with the pGBKT7 vector. The BD-VdHSFB1 and BD-VvHSFB1 plasmids were transformed into yeast strain Y2Hgold. They were transferred onto SD/−Trp/X-α-Gal/AbA and SD-Trp/-His/−Ade selection media with negative and positive controls. The primers for yeast assays are in Table S3 (see online supplementary material).

### Subcellular localization

*VdHSFB1* and *VvHSFB1* coding sequences were individually fused with the *eGFP* of the pCAMBIA2300 vector, and the resultants were co-expressed with the nuclear marker H2B-mCherry in tobacco leaves. The subcellular localization of VdHSFB1 and VvHSFB1 was detected with a Leica TCS SP5 Confocal Scanning Microscope made by Lecia (Hessen, Germany). The peak excitation wavelengths of eGFP and RFP were 488 nm and 532 nm, respectively. The primers for subcellular localization assays are in Table S3 (see online supplementary material).

### Measurement of electrolyte leakage

Electrolyte leakage was measured by the method described by Xu *et al*. with modification [64]. Ten leaves discs with 0.5 cm in diameter were incubated in 5 ml of distilled water. After shaking at 100 rpm and 25°C for 20 min, initial conductivity (C1) was determined with FE30 made by Mettler Toledo (Zurich, Switzerland). Then the leaves were boiled for 20 min. The conductivity was remeasured after shaking at 100 rpm and 25°C for 20 min as C2. The electrolyte leakage was determined by using the equation EL (%) = C1/C2 × 100.

### Statistical analysis

The statistical data analysis was performed using GraphPad Prism 8.0.1 and spss 19.0. Data was considered statistically significant at a *P*-value <0.05 using Duncan test or Student’s *t*-test. Data represent the mean ± SE from three independent experiments.

## Acknowledgements

This work was supported by the National Natural Science Foundation of China (Grant no. U21A20227), the National Key Research and Development Program of China (Grant no. 2018YFD1000300), and the Strategic Priority Research Program of the Chinese Academy of Sciences (Grant no. XDA23080602). Research conducted as part of the LIA INNOGRAPE International Associated Laboratory.

## Author contributions

H.C. performed the work and initiated the draft. X.L., S.L., and H.M. participated in the plant material screening, treatment, and collection. Y.W. participated in data analysis. Y.L. helped to do the transformation of grape plants and cells. L.Y., W.D., P.F., and Z.L. helped to design the experiment. L.W. obtained funding and designed experiments. L.Y. and L.W. did the final editing.

## Data availability

The raw data of RNA-sequencing for this article have been deposited in National Genomics Data Center (https://ngdc.cncb.ac.cn/). The BioProject accession is PRJCA009515. And the data used to support the findings of this study are available from the corresponding authors upon reasonable request.

## Conflict of interest

The authors have no conflicts of interest to declare.

## Supplementary data

Supplementary data is available at *Horticulture Research* online.

## Supplementary Material

Web_Material_uhad001
