# Supplementary material for: The class B heat shock factor HSFB1 regulates heat tolerance in grapevine
Source: Hortic Res. 2023 Jan 4;10(3):uhad001. doi: 10.1093/hr/uhad001 (PMC10018785; doi:10.1093/hr/uhad001)
Supplement: Web_Material_uhad001 [file web_material_uhad001.zip › Supplementary material.docx]

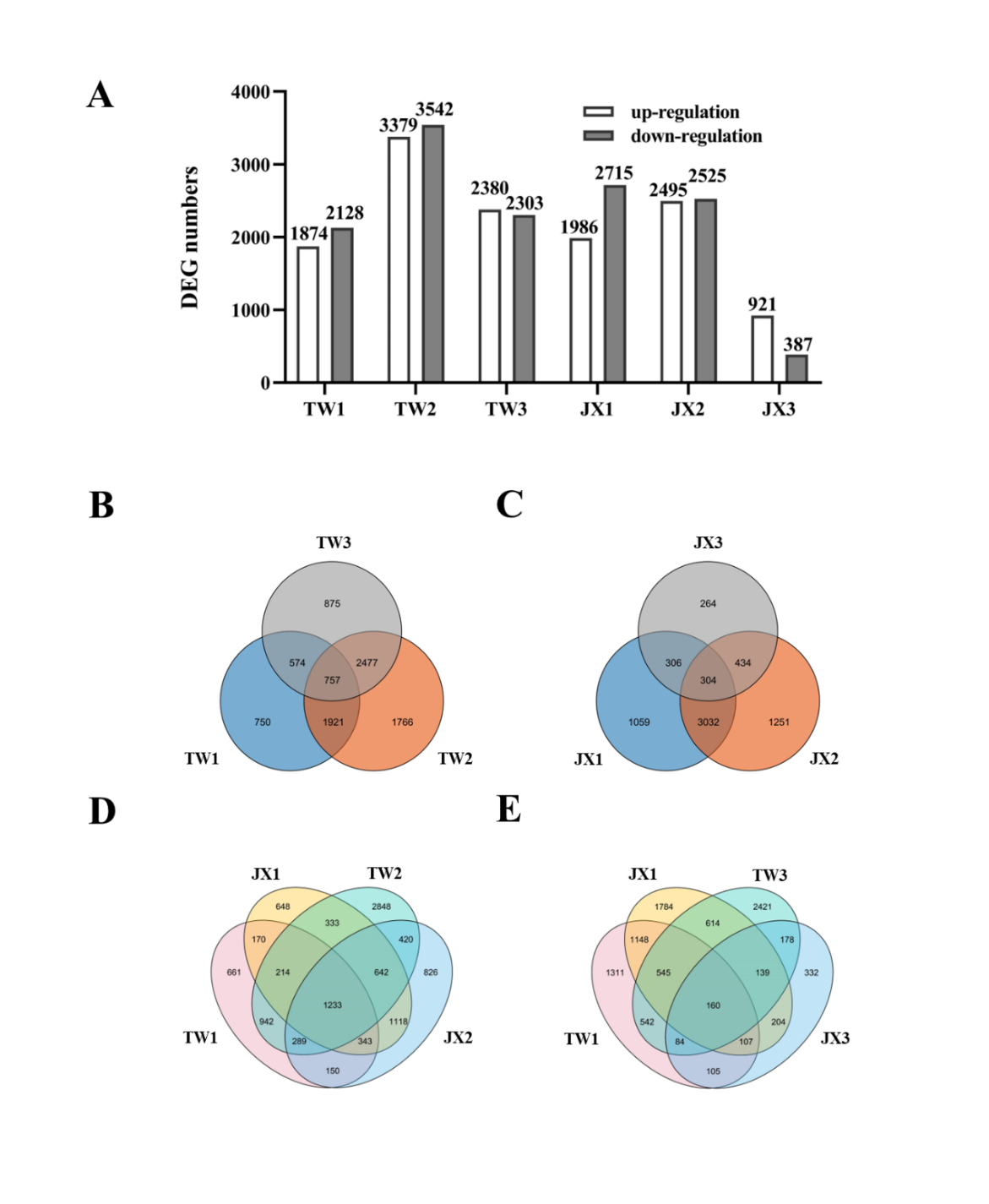


**Fig. S1** The analysis of DEGs in *V. davidii* ‘Tangwei’ and *V. vinifera* ‘Jingxiu’ under various temperature conditions (25°C, 40°C and 45°C). **A** Up- and down-regulated DEGs in ‘Tangwei’ and ‘Jingxiu’; **B-E** Venn diagrams analysis of DEGs in ‘Tangwei’ and ‘Jingxiu’. **B-C** The comparison of DEGs in ‘Tangwei’ or ‘Jingxiu’ among different temperatures. **D-E** The comparison of DEGs between ‘Tangwei’ and ‘Jingxiu’ under different temperatures. TW1 represents the DEGs comparison between the treatment of 40°C and the control of 25°C in ‘Tangwei’; TW2 and TW3 represents 40°C vs 25°C and 45°C vs 40°C, respectively. JX1 represents the DEGs comparison between the treatment of 40°C and the control of 25°C in ‘Jingxiu’. JX2 and JX3 represents 45°C vs 25°C and 45°C vs 40°C, respectively.


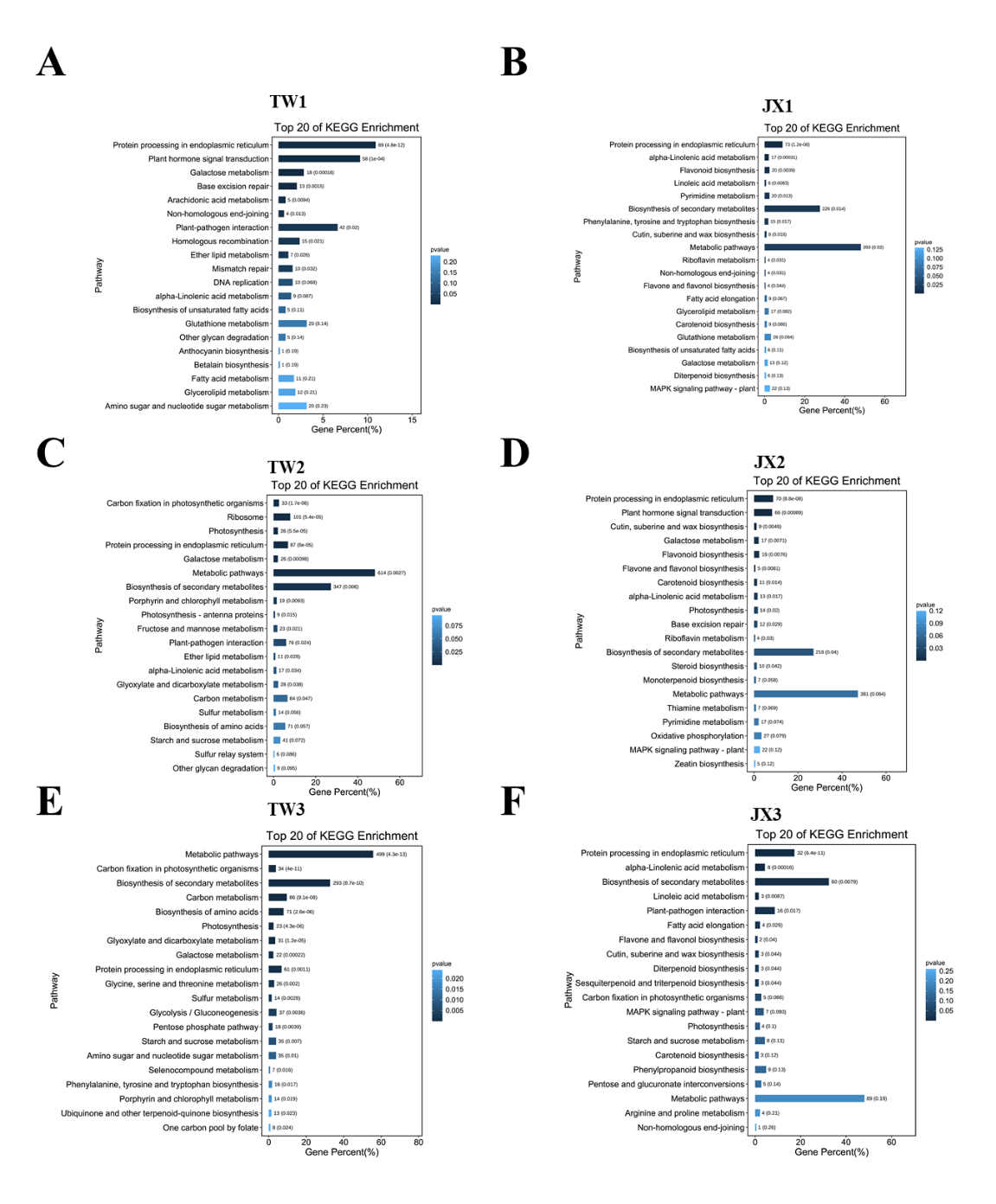


**Fig. S2** KEGG enrichment of DEGs in *V. davidii* ‘Tangwei’ and *V. vinifera* ‘Jingxiu’ under different temperature conditions (25°C, 40°C and 45°C). **A, C, E** KEGG pathway enrichment of DEGs in ‘Tangwei’ under various temperature conditions. **B, D, F** KEGG pathway enrichment of DEGs in ‘Jingxiu’ under various temperature conditions. TW1, TW2, TW3, JX1, JX2 and JX3 indicate as shown in Fig. S1.


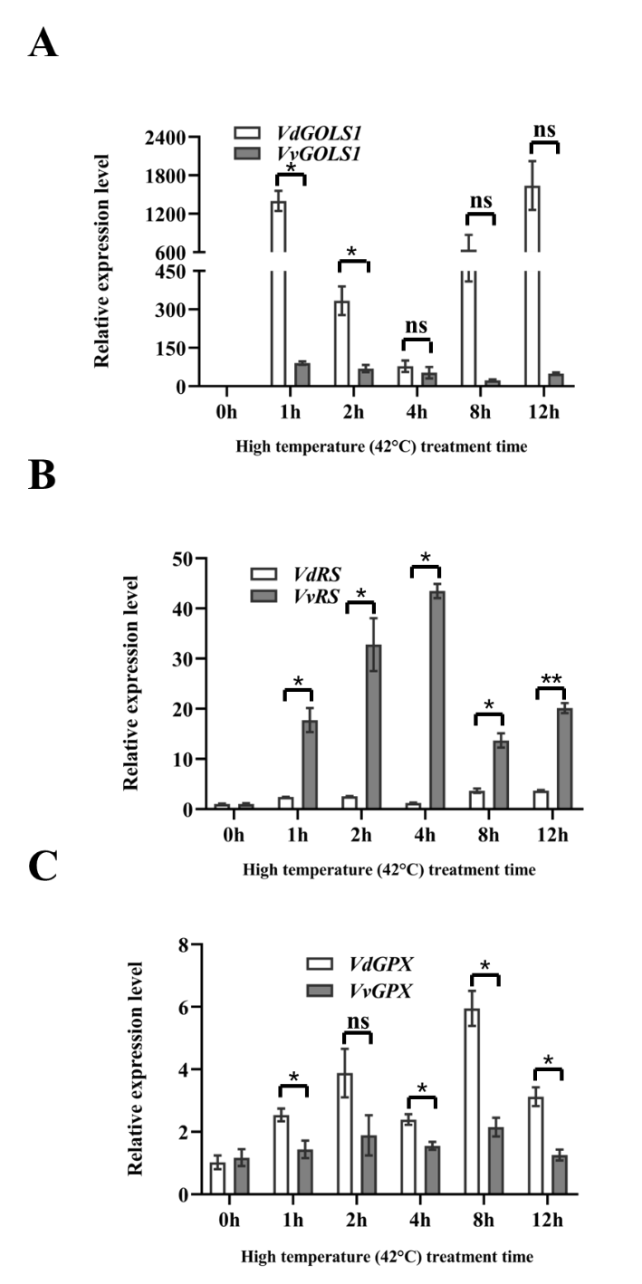


**Fig.** **S3** Expression of *GOLS1* (galactinol synthase 1), *RS* (raffinose synthase) and *GPX* (glutathione peroxidase) in *V. davidii* (*VdGOLS1*, *VdRS*, *VdGPX*) and *V. vinifera* (*VvGOLS1*, *VvRS*, *VvGPX*) under 42°C for different treatment time. **A-C** Analysis of the *GOLS1, RS* and *GPX* expression in grapevine leaves of ‘Tangwei’ and ‘Jingxiu’ under 42°C for 0, 1, 2, 4, 8 and 12 h, respectively. Data represent the mean ± SE of three biological replicates. Significant differences were determined using Student’s *t*-test: ******P* < 0.05; *******P*< 0.01.


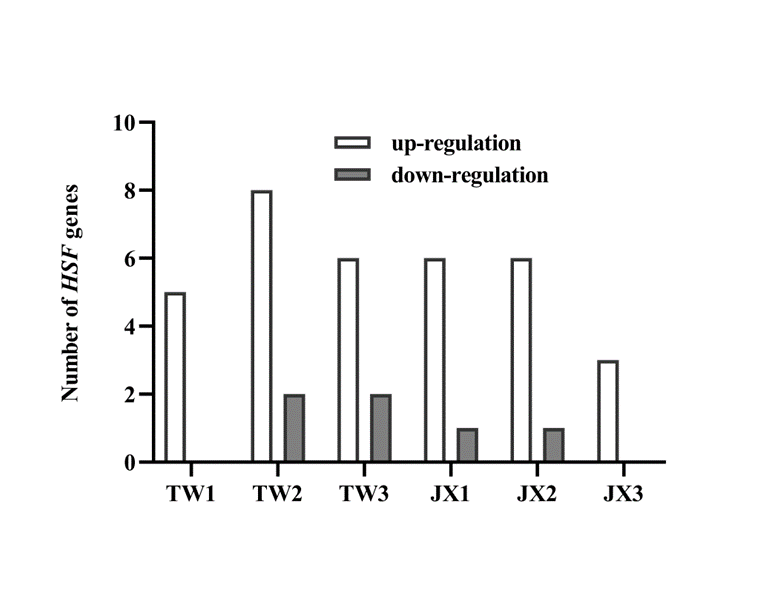


**Fig. S4** The number of DEGs (up-regulation and down-regulation) encoding HSF transcription factors in *V. davidii* ‘Tangwei’ and *V. vinifera* ‘Jingxiu’ under various temperature conditions (25°C, 40°C and 45°C). TW1, TW2, TW3, JX1, JX2 and JX3 indicate as shown in Fig. S1.

**Fig. S5** Comparison of *HSFB1* coding sequence of *V. davidii* ‘Tangwei’ and *V. vinifera* ‘Jingxiu’. Alignment was performed using DNAMAN. The coding sequence of *HSFB1* in Pinot Noir is used as reference sequence.


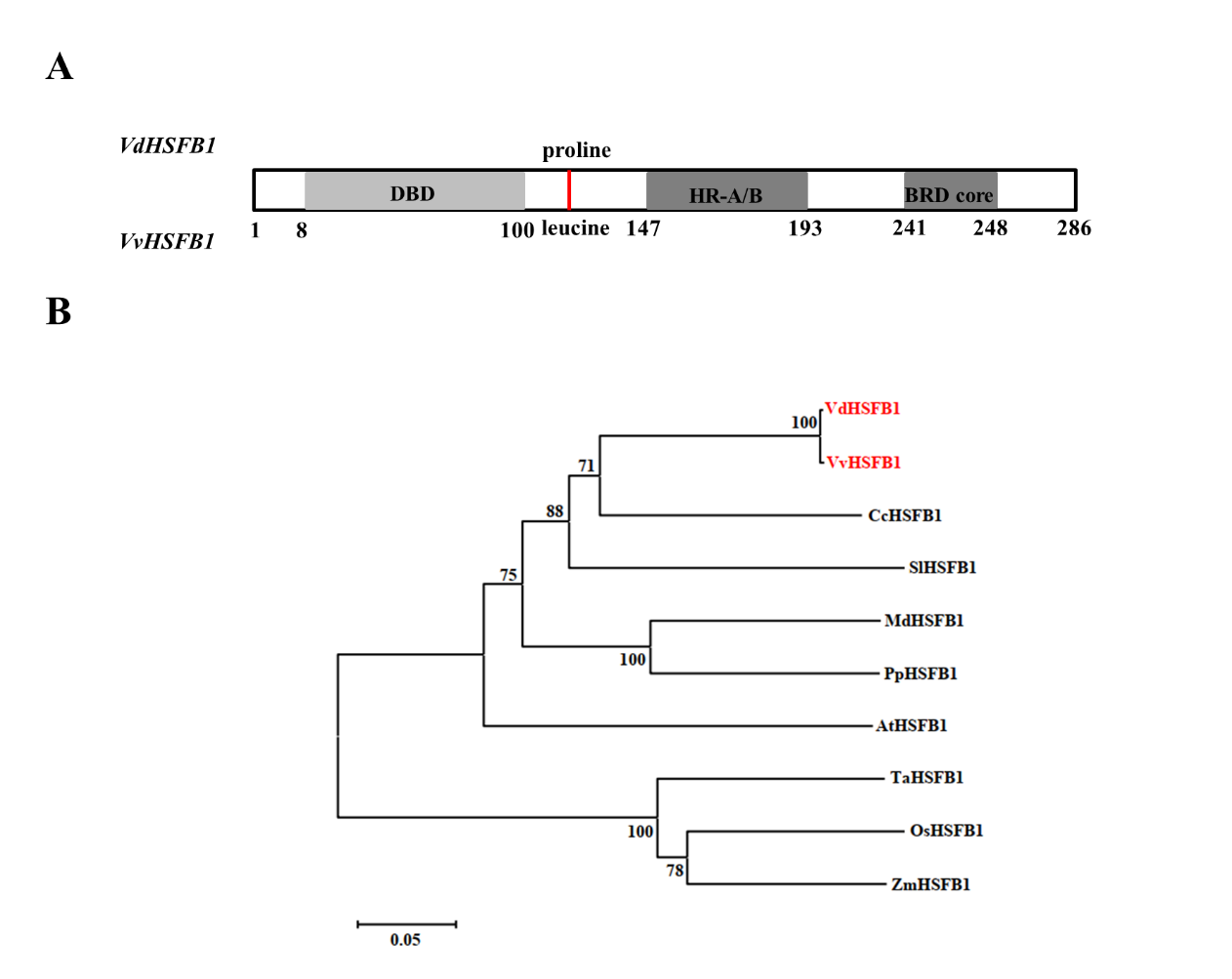


**Fig. S6** The characteristic analysis of VdHSFB1 and VvHSFB1 proteins. **A** The amino acid sequence comparison of VdHSFB1 and VvHSFB1. DBD indicates DNA binding domain; HR-A/B indicates the oligomerization domain; BRD indicates B3 repression domain; the number at the bottom of box means the position of amino acid. **B** The phylogenetic relationship between VdHSFB1, VvHSFB1 and other HSF proteins, including CcHSFB1 from *Citrus clementina*, SlHSFB1 from *Solanum lycopersicum*, MdHSFB1 from *Malus domestica*, PpHSFB1 from *Prunus persica*, AtHSFB1 from *Arabidopsis thaliana*, TaHSFB1 from *Triticum aestivum*, OsHSFB1 from *Oryza sativa*, ZmHSFB1 from *Zea mays*. The tree was conducted using MEGA-X64 and the statistical reliability of individual nodes was evaluated by bootstrap analysis with 1000 replicates.


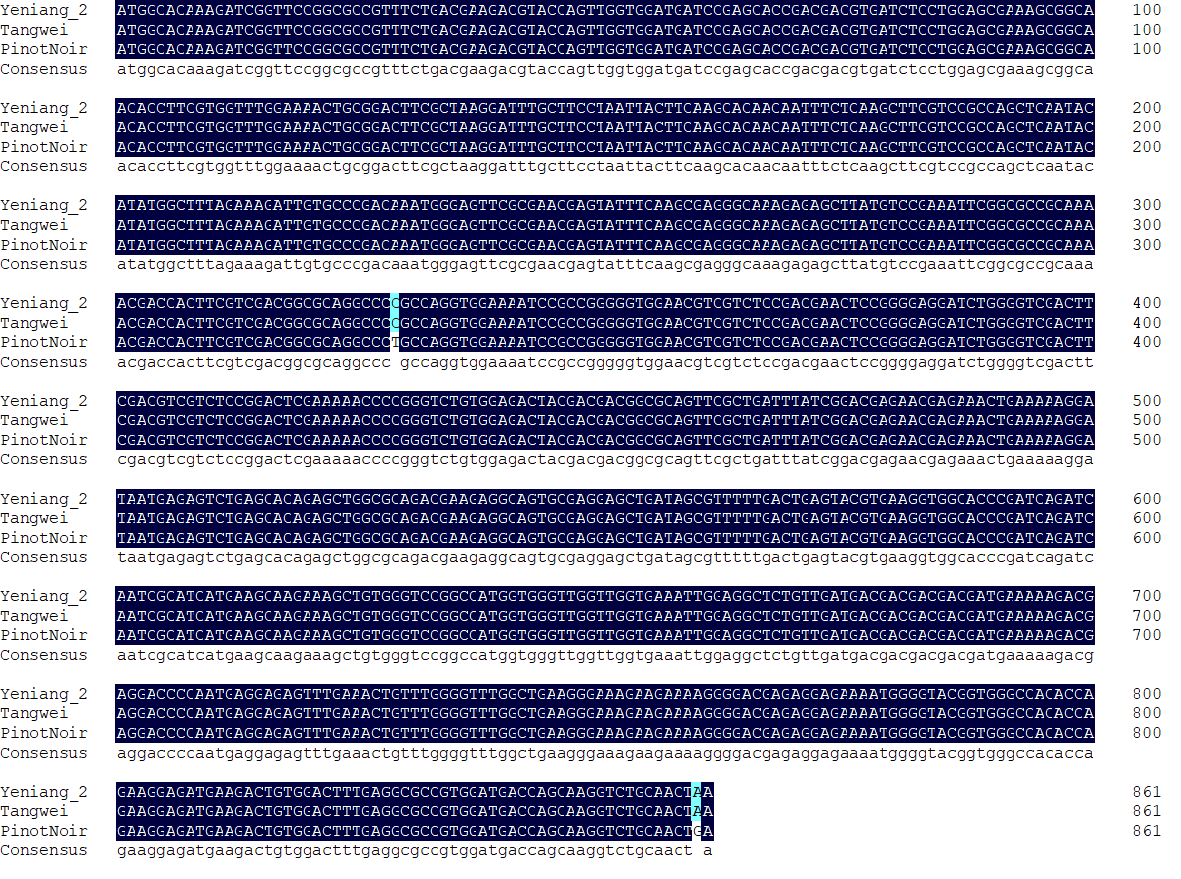


**Fig. S7** Comparison of *HSFB1* coding sequence of *V. quinquangularis* ‘Yeniang 2’ and *V. davidii* ‘Tangwei’. Alignment was performed using DNAMAN. The coding sequence of *HSFB1* in Pinot Noir is used as reference sequence.


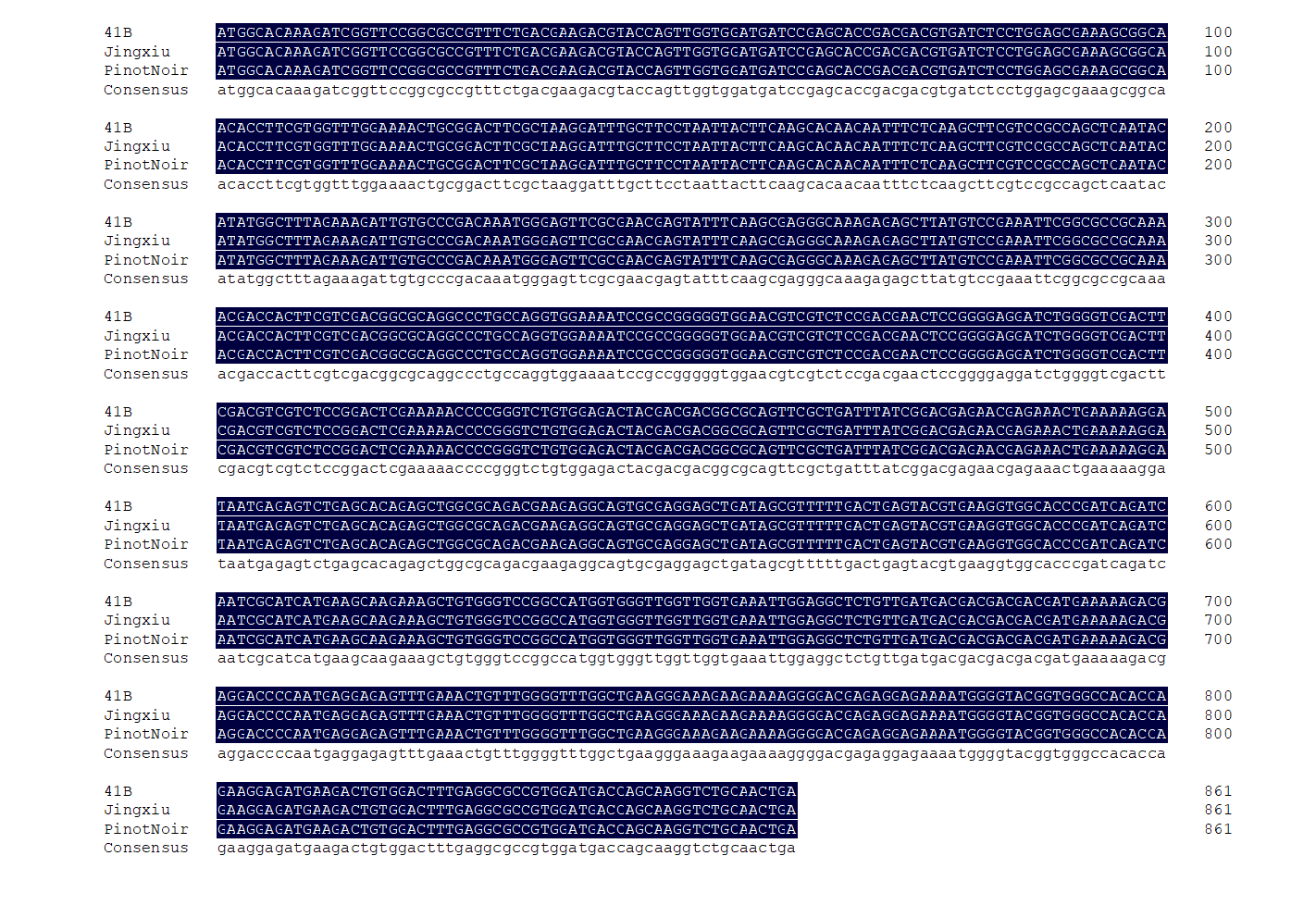


**Fig. S8** Comparison of *HSFB1* coding sequences of *V. vinifera* ‘Chasselas’ × *V. berlandieri* (‘41B’) and *V. vinifera* (‘Jingxiu’). Alignment was performed using DNAMAN. The coding sequence of *HSFB1* in Pinot Noir is used as reference sequence.

**Fig. S9** The sequence comparison between *VdHSFB1* and *VvHSFB1* promoters. The promoter sequence was cloned 2500 bp upstream from transcription start site. Alignment was performed using DNAMAN. The promoter sequence of *HSFB1* in Pinot Noir is used as reference sequence.

**Table S1** The alignment of samples sequencing data with reference genome (12X) of grape

| Samples | Total Reads | Mapped Reads | Uniq Mapped Reads | Multiple Map Reads | Reads Map to '+' | Reads Map to '-' |
| --- | --- | --- | --- | --- | --- | --- |
| T-25-1 | 48051628 | 29383834 (61.15%) | 28798224 (59.93%) | 585610 (1.22%) | 14573751 (30.33%) | 14648759 (30.49%) |
| T-25-2 | 46137206 | 28004311 (60.70%) | 27456101 (59.51%) | 548210 (1.19%) | 13891104 (30.11%) | 13949659 (30.24%) |
| T-25-3 | 53183456 | 32774635 (61.63%) | 32162460 (60.47%) | 612175 (1.15%) | 16245150 (30.55%) | 16346577 (30.74%) |
| T-40-1 | 43602960 | 26318911 (60.36%) | 25806757 (59.19%) | 512154 (1.17%) | 13064823 (29.96%) | 13127439 (30.11%) |
| T-40-2 | 44708976 | 26569276 (59.43%) | 26001991 (58.16%) | 567285 (1.27%) | 13167746 (29.45%) | 13256780 (29.65%) |
| T-40-3 | 48644130 | 29046688 (59.71%) | 28442015 (58.47%) | 604673 (1.24%) | 14404191 (29.61%) | 14488492 (29.78%) |
| T-45-1 | 55296618 | 36834424 (66.61%) | 36006902 (65.12%) | 827522 (1.50%) | 18217686 (32.95%) | 18397393 (33.27%) |
| T-45-2 | 49440224 | 32348759 (65.43%) | 31447365 (63.61%) | 901394 (1.82%) | 15959124 (32.28%) | 16086655 (32.54%) |
| T-45-3 | 50998992 | 33324744 (65.34%) | 32202672 (63.14%) | 1122072 (2.20%) | 16386792 (32.13%) | 16543690 (32.44%) |
| J-25-1 | 43836276 | 29366059 (66.99%) | 28933585 (66.00%) | 432474 (0.99%) | 14637012 (33.39%) | 14650957 (33.42%) |
| J-25-2 | 51677964 | 34639064 (67.03%) | 34104099 (65.99%) | 534965 (1.04%) | 17270614 (33.42%) | 17275845 (33.43%) |
| J-25-3 | 51070424 | 33073279 (64.76%) | 32576577 (63.79%) | 496702 (0.97%) | 16500063 (32.31%) | 16485100 (32.28%) |
| J-40-1 | 50341012 | 31931057 (63.43%) | 31171363 (61.92%) | 759694 (1.51%) | 15843960 (31.47%) | 15831559 (31.45%) |
| J-40-2 | 49284518 | 32234169 (65.40%) | 31677579 (64.27%) | 556590 (1.13%) | 16044947 (32.56%) | 16069902 (32.61%) |
| J-40-3 | 44495506 | 28830288 (64.79%) | 28159556 (63.29%) | 670732 (1.51%) | 14291080 (32.12%) | 14334778 (32.22%) |
| J-45-1 | 47335692 | 29144801 (61.57%) | 28162520 (59.50%) | 982281 (2.08%) | 14382921 (30.38%) | 14401854 (30.42%) |
| J-45-2 | 44291866 | 26071169 (58.86%) | 25581600 (57.76%) | 489569 (1.11%) | 12991063 (29.33%) | 12955281 (29.25%) |
| J-45-3 | 51918196 | 32181444 (61.98%) | 31245073 (60.18%) | 936371 (1.80%) | 15901166 (30.63%) | 15945877 (30.71%) |

T-25 represents ‘Tangwei’ grapevine were treated under 25°C for 2 h; T-40 and T-45 represents ‘Tangwei’ grapevine were treated under 40°C and 45°Cfor 2 h, respectively; J-25 represents ‘Jingxiu’ grapevine were treated under 25°C for 2 h; J-40 and J-45 represents ‘Jingxiu’ grapevine were treated under 40°C and 45°Cfor 2 h, respectively.

**Table S2** The comparison of *VdHSFB1* and *VvHSFB1* promoters in cis-elements

| the name of cis-element | Number | |
| --- | --- | --- |
|  | *VdHSFB1* promoter | *VvHSFB1* promoter |
| AAGAA-motif | 2 | 2 |
| ABRE | 1 | 1 |
| AC-I | 1 | 1 |
| AC-II | 1 | 1 |
| **ARE** | 7 | 6 |
| ATW1-motif | 1 | 1 |
| **AT~TATA-box** | 4 | 2 |
| Box 4 | 2 | 3 |
| CAAT-box | 41 | 42 |
| CGTCA-motif | 1 | 1 |
| ERE | 5 | 5 |
| G-Box | 1 | 1 |
| GATA-motif | 1 | 1 |
| GCN4_motif | 1 | 1 |
| GTW1-motif | 2 | 2 |
| HD-Zip 1 | 1 | 1 |
| LAMP-element | 1 | 1 |
| LS7 | 1 | 1 |
| **MYB** | 4 | 3 |
| MYB-like sequence | 2 | 2 |
| MYC | 3 | 3 |
| Myb | 1 | 1 |
| P-box | 1 | 1 |
| STRE | 1 | 1 |
| Sp1 | 1 | 1 |
| **TATA-box** | 57 | 47 |
| TC-rich repeats | 1 | 1 |
| TGACG-motif | 1 | 1 |
| WRE3 | 2 | 2 |
| as-1 | 1 | 1 |
| box S | 1 | 1 |
| circadian | 1 | 1 |

**Table S3** The primers used in this study

| Purpose | Primer name | Sequences (5'To3') |
| --- | --- | --- |
| qRT-PCR analysis | VvACTIN7-F | CTTGCATCCCTCAGCACCTT |
|  | VvACTIN7-R | TCCTGTGGACAATGGATGGA |
|  | HSFB1-qPCR-F | ACTGAGTACGTGAAGGTGGC |
|  | HSFB1-qPCR-R | TCGTCGTCATCAACAGAGCC |
|  | RS-qPCR-F | AACACTACGCCCTTCCCTCA |
|  | RS-qPCR-R | GAGGCGCTCTTGTTTCTTCG |
|  | GOLS1-qPCR-F | AACGGGGACTACGTGAAAGG |
|  | GOLS1-qPCR-R | TGGTTCTCGGGTGGGTAAAC |
|  | GPX-qPCR-F | TCACCGTTAAGGATGCTGAGG |
|  | GPX-qPCR-R | GGCCTTGATCTTTGTACTTCTCG |
| Promoter and gene isolation | HSFB1-promoter-F | AATGGATCATCCCACAAAGTGA |
|  | HSFB1-promoter-R | TTCTCCCCGATTTGTCCCTCAC |
|  | HSFB1-CDS-F | ATGGCACAAAGATCGGTTCCGG |
|  | HSFB1-CDS-R | TCAGTTGCAGACCTTGCTGGTC |
| Subcellular localization and transformation | HSFB1-2300-F | TCATTTGGAGAGAACACGGGGGACGAGCTCGGTACCATGGCACAAAGATCGGTTCCGG |
|  | HSFB1-2300-R | GCCCTTGCTCACCATGGTGTCGACTCTAGAGGATCCGTTGCAGACCTTGCTGGTCATC |
|  | 2300-F | ACTATCCTTCGCAAGACCCT |
|  | 2300-R | CAGGGTCAGCTTGCCGTAG |
|  | SiHSFB1-5941-F | CGGGCGCGCCGGATCCCTTCGTCCGCCAGCTCAATA |
|  | SiHSFB1-5941-R | GCCCATGGTCTAGACGTCGTAGTCTCCACAGACC |
|  | SiHSFB1-check-F | CTTCGTCCGCCAGCTCAATA |
|  | SiHSFB1-check-R | CGTCGTAGTCTCCACAGACC |
| Yeast assays | HSFB1-PGBKT7-F | GGAGCAGAAGCTGATCTCAGAGGAGGACCTGCATATGATGGCACAAAGATCGGTTCCG |
|  | HSFB1-PGBKT7-R | GCGGCCGCTGCAGGTCGACGGATCCCCGGGAATTCTCAGTTGCAGACCTTGCTGGTCA |
| Transient luciferase expression assay | HSFB1-GAL4BD-F | TTGACTGTATCGCCGTCTAGAACTAGTGGATCCATGGCACAAAGATCGGTTCCG |
|  | HSFB1-GAL4BD-R | GTCGACGGTATCGATAAGCTTGATATCGAATTCTTAGTTGCAGACCTTGCTGGT |
|  | HSFB1-0800luc-F | GTAATACGACTCACTATAGGGCGAATTGGGTACCAATGGATCATCCCACAAAGTGA |
|  | HSFB1-0800luc-R | CGCTCTAGAACTAGTGGATCCCCCGGGCTGCAGTTCTCCCCGATTTGTCCCTCAC |
