# Supplementary figures and images for: The class B heat shock factor HSFB1 regulates heat tolerance in grapevine
Source: Hortic Res. 2023 Jan 4;10(3):uhad001. doi: 10.1093/hr/uhad001 (PMC10018785; doi:10.1093/hr/uhad001)

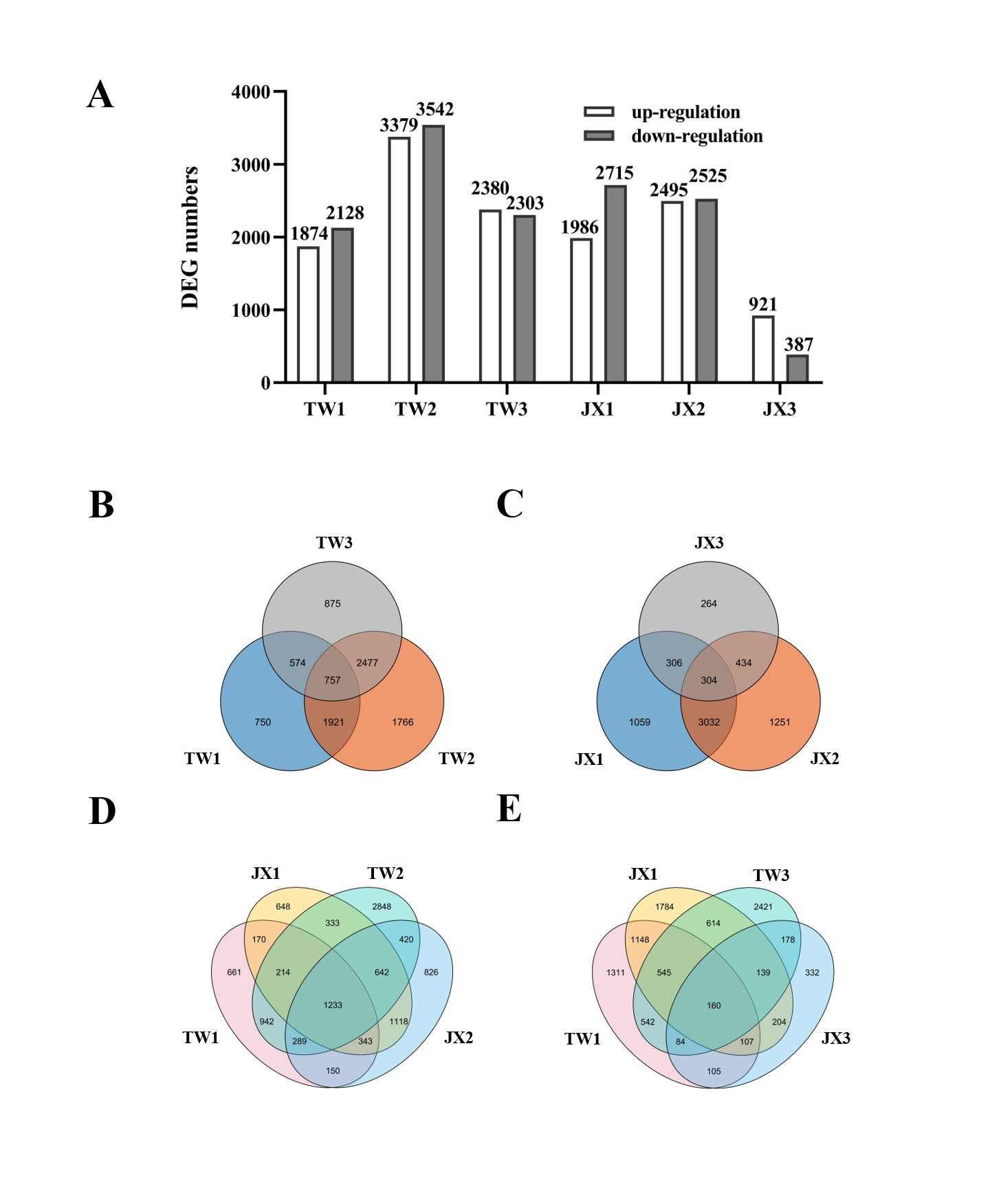

Supplement: Web_Material_uhad001 [file web_material_uhad001.zip › Fig.S1.png]

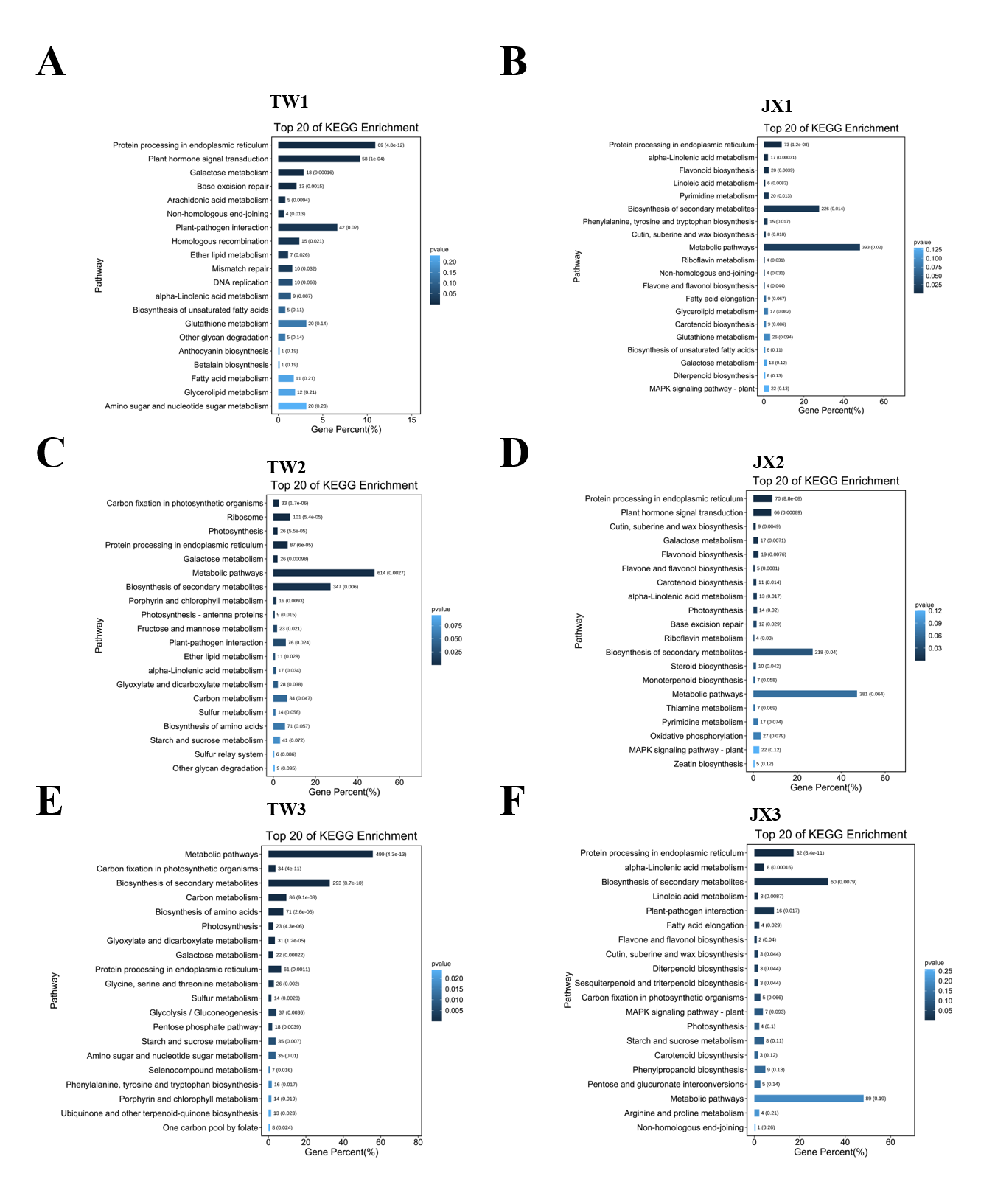

Supplement: Web_Material_uhad001 [file web_material_uhad001.zip › Fig.S2.png]

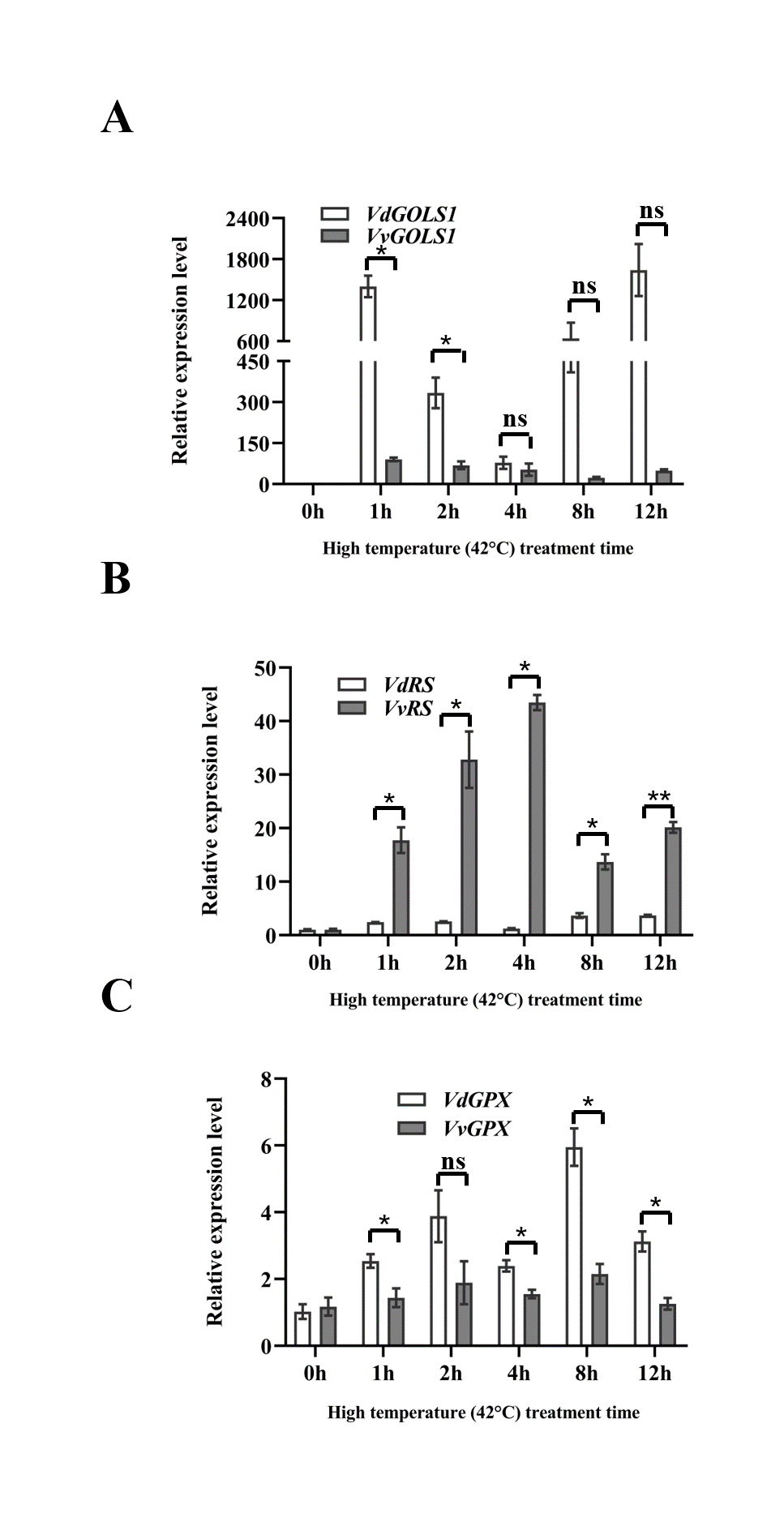

Supplement: Web_Material_uhad001 [file web_material_uhad001.zip › Fig.S3.png]

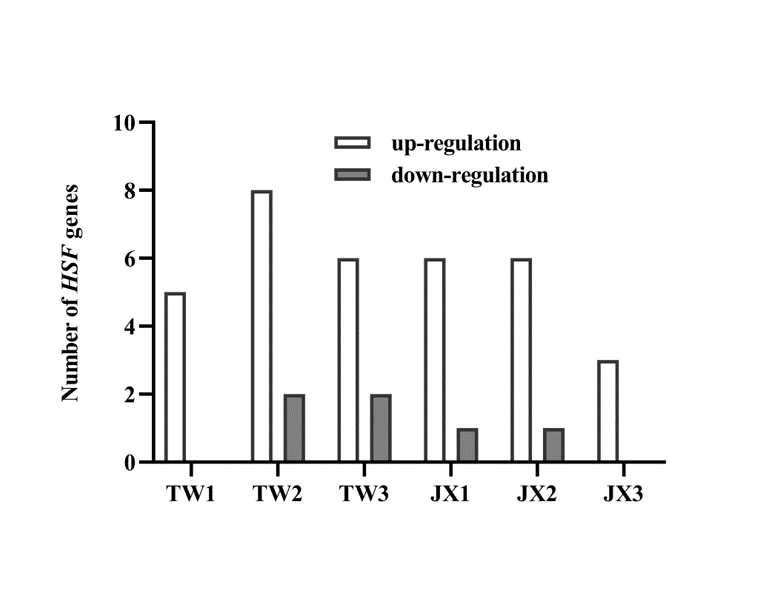

Supplement: Web_Material_uhad001 [file web_material_uhad001.zip › Fig.S4.png]

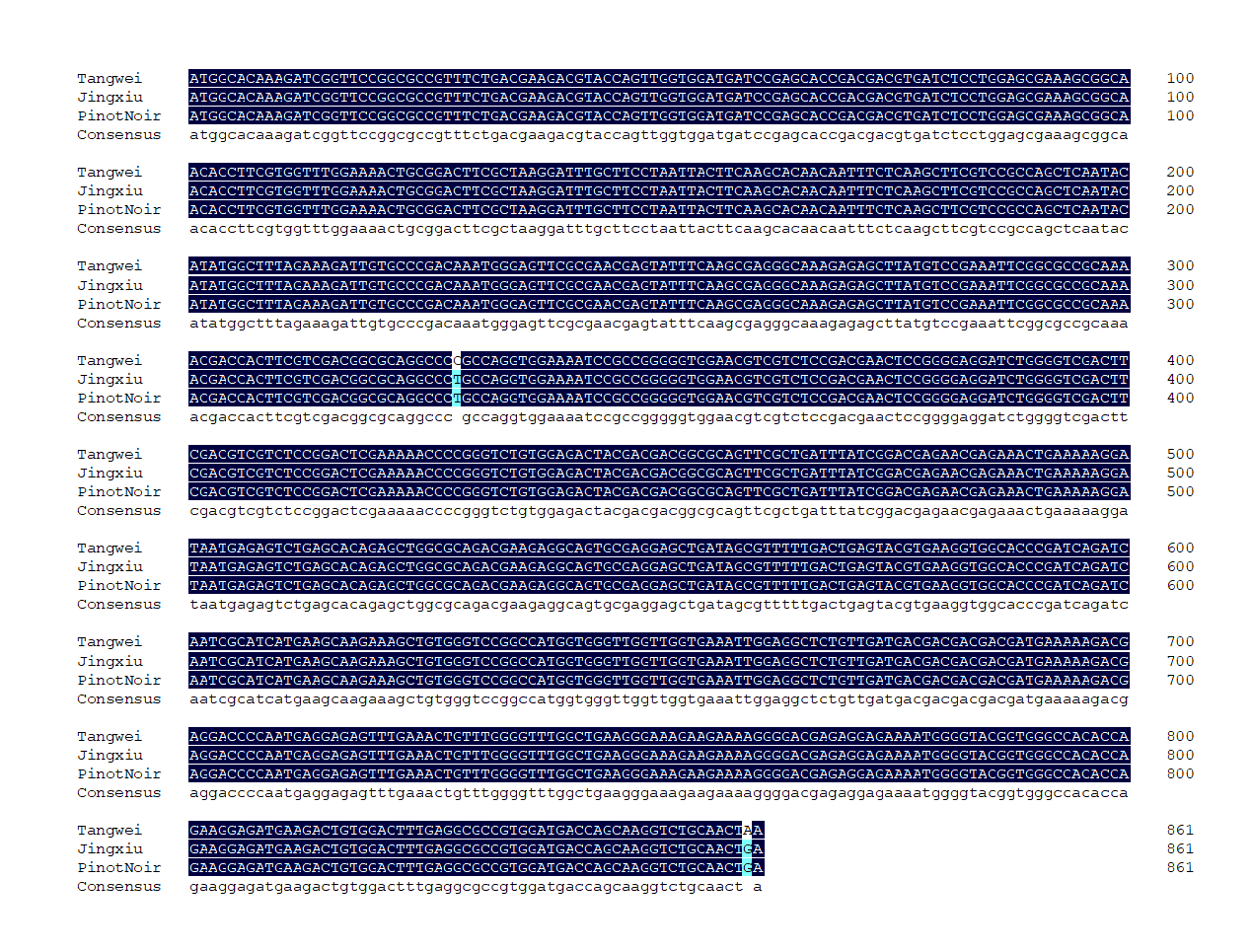

Supplement: Web_Material_uhad001 [file web_material_uhad001.zip › Fig.S5.png]

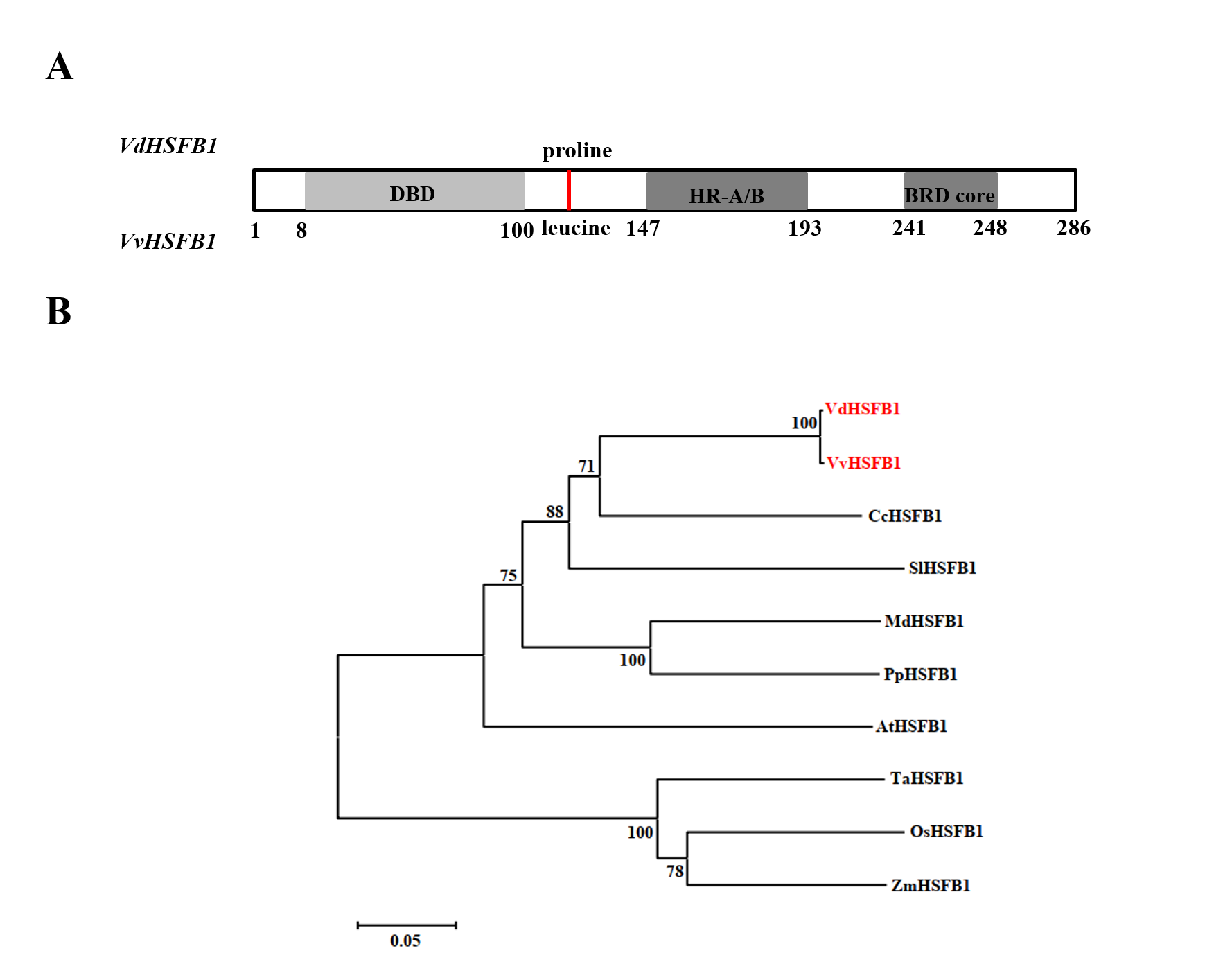

Supplement: Web_Material_uhad001 [file web_material_uhad001.zip › Fig.S6.png]

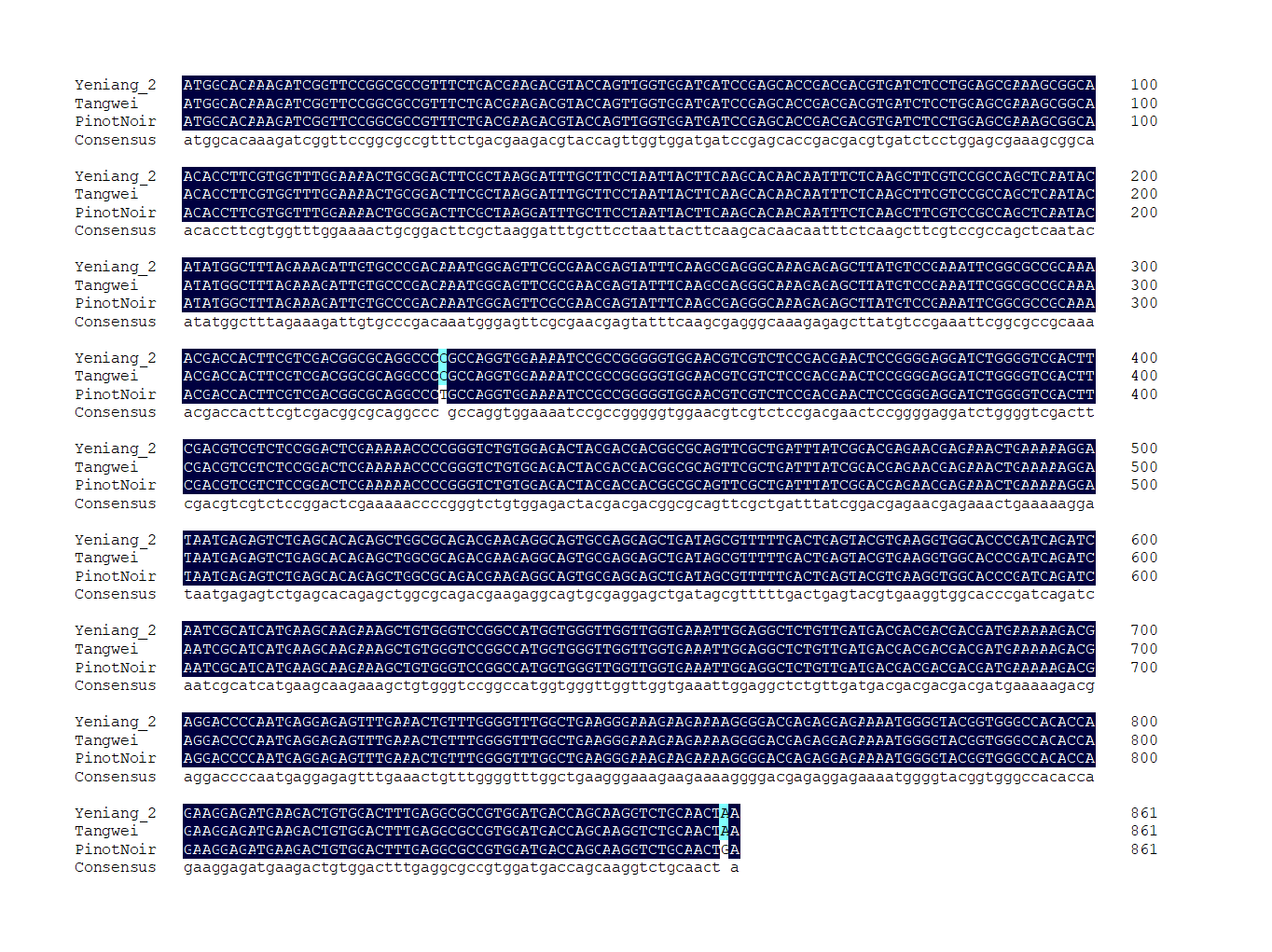

Supplement: Web_Material_uhad001 [file web_material_uhad001.zip › Fig.S7.png]

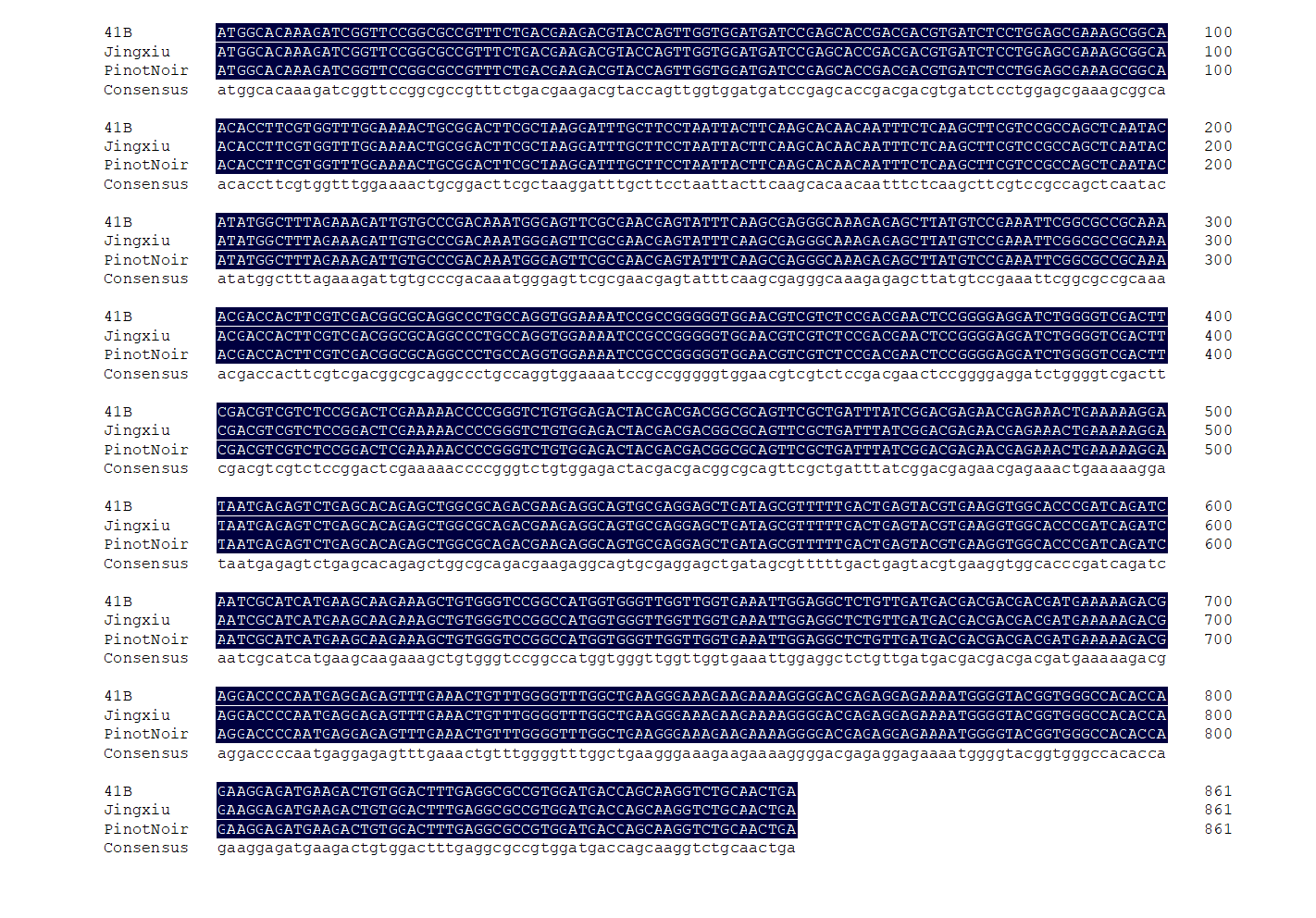

Supplement: Web_Material_uhad001 [file web_material_uhad001.zip › Fig.S8.png]

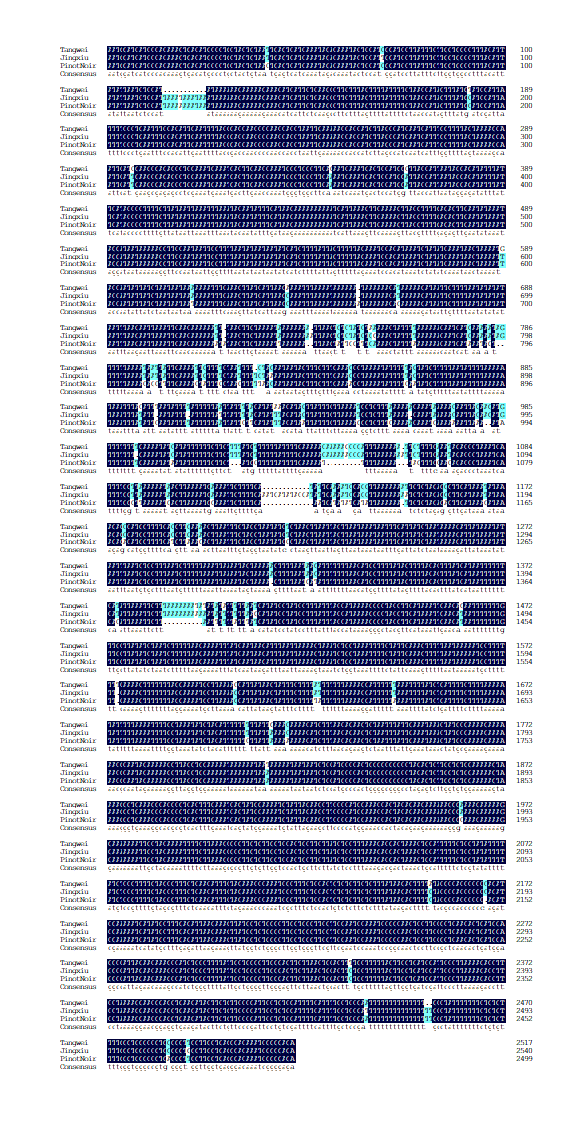

Supplement: Web_Material_uhad001 [file web_material_uhad001.zip › Fig.S9.png]

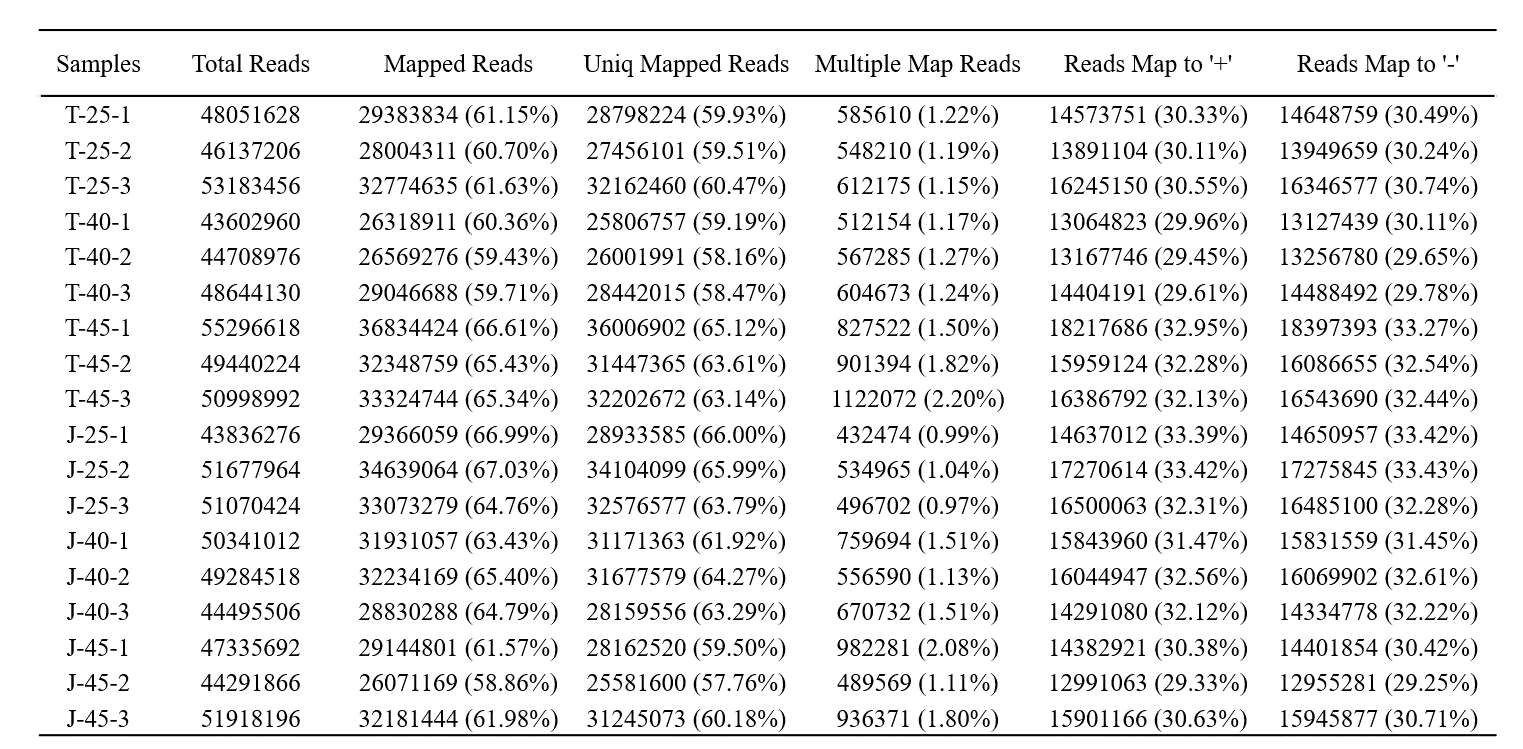

Supplement: Web_Material_uhad001 [file web_material_uhad001.zip › Table S1.png]

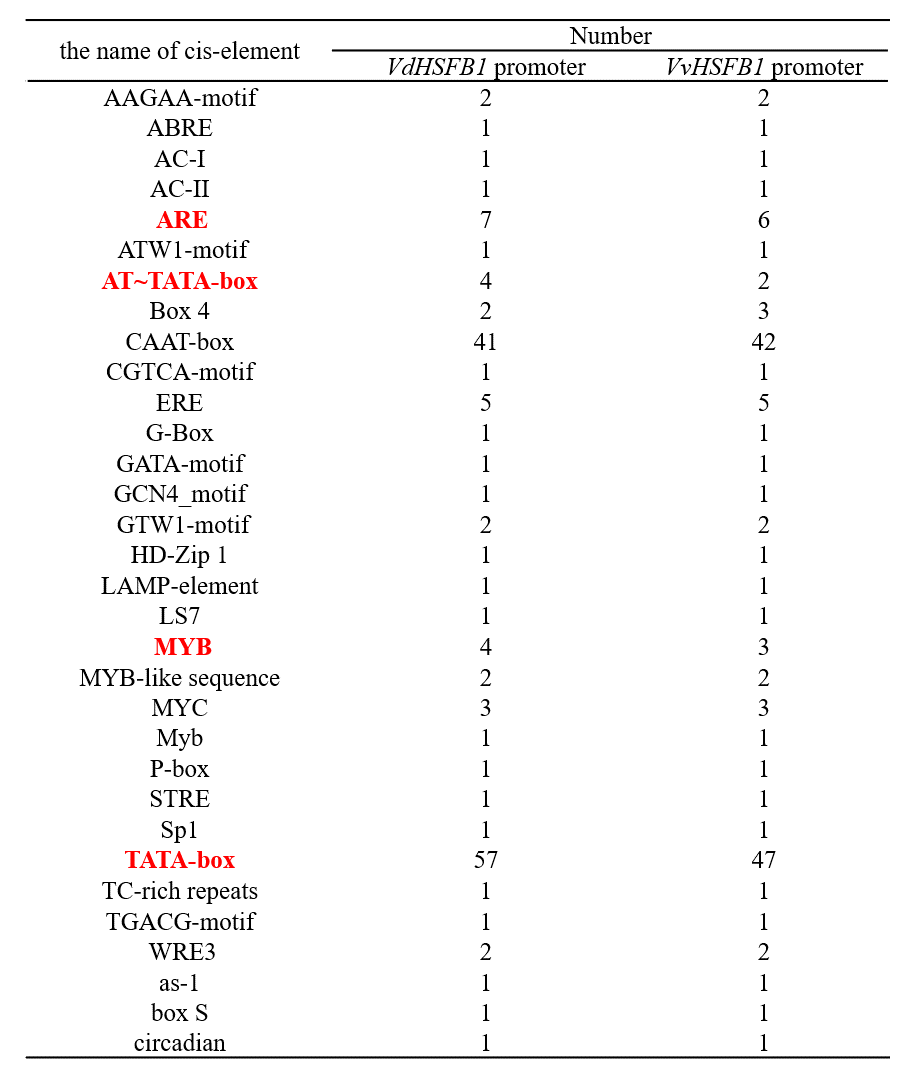

Supplement: Web_Material_uhad001 [file web_material_uhad001.zip › Table S2.png]

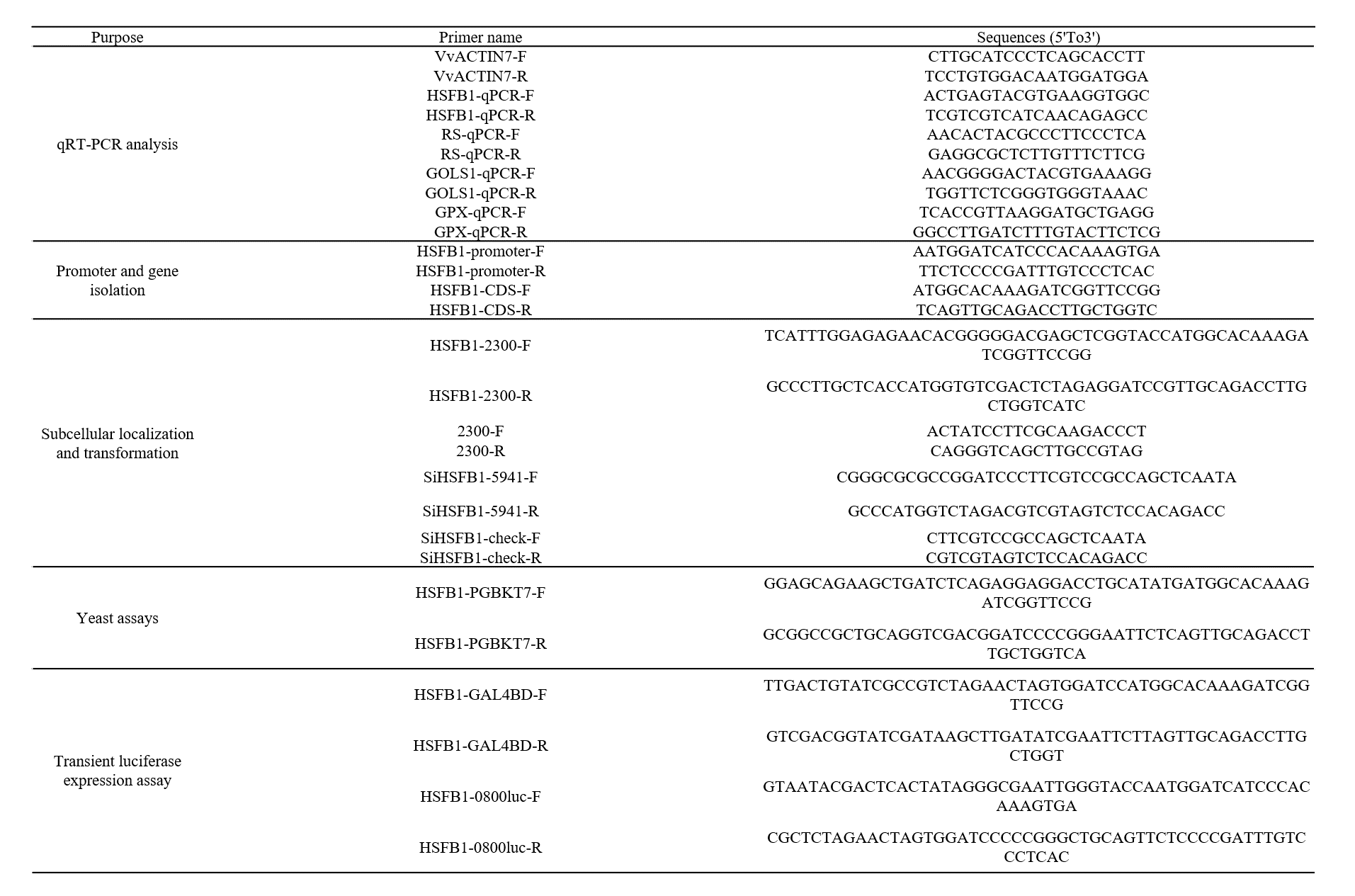

Supplement: Web_Material_uhad001 [file web_material_uhad001.zip › Table S3.png]
